# Functional diversity and nutritional content in a deep-sea faunal assemblage through total lipid, lipid class, and fatty acid analyses

**DOI:** 10.1371/journal.pone.0207395

**Published:** 2018-11-12

**Authors:** Camilla Parzanini, Christopher C. Parrish, Jean-François Hamel, Annie Mercier

**Affiliations:** 1 Department of Ocean Sciences, Memorial University, St. John’s, NL, Canada; 2 Society for Exploration and Valuing of the Environment (SEVE), Portugal Cove-St. Philips, NL, Canada; University of Illinois, UNITED STATES

## Abstract

Lipids are key compounds in marine ecosystems being involved in organism growth, reproduction, and survival. Despite their biological significance and ease of measurement, the use of lipids in deep-sea studies is limited, as is our understanding of energy and nutrient flows in the deep ocean. Here, a comprehensive analysis of total lipid content, and lipid class and fatty acid composition, was used to explore functional diversity and nutritional content within a deep-sea faunal assemblage comprising 139 species from 8 phyla, including the Chordata, Arthropoda, and Cnidaria. A wide range of total lipid content and lipid class composition suggested a diversified set of energy allocation strategies across taxa. Overall, phospholipid was the dominant lipid class. While triacylglycerol was present in most taxa as the main form of energy storage, a few crustaceans, fish, jellyfishes, and corals had higher levels of wax esters/steryl esters instead. Type and amount of energy reserves may reflect dietary sources and environmental conditions for certain deep-sea taxa. Conversely, the composition of fatty acids was less diverse than that of lipid class composition, and large proportions of unsaturated fatty acids were detected, consistent with the growing literature on cold-water species. In addition, levels of unsaturation increased with depth, likely suggesting an adaptive strategy to maintain normal membrane structure and function in species found in deeper waters. Although proportions of n-3 fatty acids were high across all phyla, representatives of the Chordata and Arthropoda were the main reservoirs of these essential nutrients, thus suggesting health benefits to their consumers.

## Introduction

Lipids represent the densest form of energy in marine ecosystems since they provide about 1.5 and 2 times more energy per gram than proteins and carbohydrates, respectively [1, 2]. They are also key components of cell membranes [1], and are involved in numerous cellular and physiological processes crucial to the reproduction, growth, and general survival of organisms [2, 3]. For example, lipids are deposited during oogenesis in fish and zooplankton [1, 2], and several other marine taxa [1, 2], and they can be transferred as lipoprotein from mother to oocytes to provide energy to embryos [4].

By definition, lipids are insoluble in polar solvents, but soluble in non-polar organic solvents which makes them relatively easy to extract from biological tissues for analysis [5]. For this reason, they have become useful in investigations of the drivers of ecosystem health and functioning [6], food web structure and dynamics [7], as well as carbon cycling [8] and contaminant bioaccumulation [9] in the marine environment. While a vast body of literature exists for shallow-water species [10–14], the study of lipids in deep-sea taxa lags behind, and is mostly limited to the analysis of fatty acids as trophic biomarkers [15–17] with a focus on certain deep-water taxa or faunal groups, such as fish, corals, and zooplankton [18–21].

Lipid extracts of aquatic samples can be separated into different classes, including phospholipids (PL) and triacylglycerols (TAG), which are of primary interest in studies of marine ecosystems [1]. Specifically, PL are the principal constituents of animal cell membranes and are found in all animal phyla [3, 6]; while TAG are the main form of energy storage in both terrestrial and marine animals [6]. Other lipid classes, such as sterols (ST) and wax esters (WE), also play important roles in marine organisms. ST are key constituents of animal cell surface membranes [22]. They are also precursors of steroid hormones, and represent essential dietary nutrients for bivalves [23], crustaceans [23] and other marine taxa [1]. Conversely, WE constitute the primary energy storage of certain shallow-water corals and sea anemones [24], as well as deep-sea crustaceans and fish [16, 21]. Wax esters also control buoyancy in myctophid fish [25] and diapausing zooplankton which overwinter in deep waters and re-enter the surface layers in spring to feed [21]. Not only single lipid classes, but also lipid class composition provides useful information about the biology and ecology of organisms. For example, the triacylglycerol to sterol ratio (TAG:ST) can assess the physiological condition of fish, bivalve, and crustacean larvae exposed to various stressors [26]; and the phospholipid to sterol ratio (PL:ST) provides an indication of membrane fluidity in fish and bivalves [21].

As major components of most lipids, fatty acids (FA) are commonly referred to as “building blocks” [27, 28]. Two FA chains (or acyl chains) are for instance attached to the glycerol backbone of a PL molecule, whereas TAG is comprised of three FA chains. Dietary FA can be either oxidized to produce high-energy molecules (i.e. ATP), or they can be transferred into membrane PL, where they play a major role in membrane structure and function [3]. In addition, certain FA are considered essential nutrients because required for optimal health and most organisms are unable to synthesize them *de novo* [5, 27, 28]. In marine ecosystems, three major essential FA can be identified, including docosahexaenoic (DHA; 22:6n-3) and eicosapentaenoic (EPA; 20:5n-3) acids from the n-3 series, and arachidonic acid (ARA; 20:4n-6) from the n-6 series. These specific polyunsaturated FA (PUFA) are precursors of docosanoids and eicosanoids, which regulate numerous cell processes [5]. Through biochemical and biophysical processes, 22:6n-3, 20:5n-3, and 20:4n-6 are involved in neurological development and signaling [29], and support immunity [30] and growth [5]. However, the extent to which these three essential FA are required and occur within tissues may vary across taxa, or even intraspecifically with age, sex, season, and habitat [28, 31, 32]. Typically, marine organisms present higher levels of n-3 PUFA than terrestrial counterparts, which instead have larger proportions of n-6 PUFA [28]. While a latitudinal trend has been found, whereby marine species from polar regions have higher levels of PUFA than those from tropical areas [28], a limited number of studies has compared shallow and deep-water species. Stowasser et al. [33] observed that shallower (<4000 m) individuals of deep-sea macrourid and morid fish species, collected in the Northeast Atlantic, had higher proportions of PUFA in their liver than their deeper counterparts. Conversely, monounsaturated FA (MUFA) increased with depth, while no bathymetric trends were detected for either PUFA or MUFA when analyzing muscle tissue [33].

The Canadian province of Newfoundland and Labrador is located in a cold-temperate region of the Northwest Atlantic, where species with subarctic/Arctic affinities are common. While several studies have been carried out in coastal and other shallow-water ecosystems of the region [11–13, 34–39], information on the lipid content and composition of the deep-sea counterparts remains fragmentary. Data only exist for total lipid contents and classes (50–1500 m) [19], as well as FA composition in corals (770–1370 m) [40]; and lipid contents, classes, and FA signatures in deep-sea gastropods and their epibiotic sea anemones (191–627 m) [41]. In order to provide novel information and baseline data for a broader range of deep dwelling taxa, the present investigation assessed total lipid content, lipid classes, and FA composition inside a deep-sea macrofaunal assemblage sampled within a tight temporal and spatial window in the Northwest Atlantic. The rich diversity analyzed here included 139 species across 8 major phyla, collected on the upper and mid-slope area off the east coast of Newfoundland. We explored the lipid profiles of a wide range of deep-sea taxa, most of which have not been studied in these terms yet, such as the Ascidiacea, and conducted both a broad cross-taxa comparative analysis, and an in-depth phylum-specific study of selected lipid and FA groups indicative of energy-storage strategies, physiological processes and dietary value for consumers, including humans. High levels of variability in lipid class and FA compositions were expected to occur within and across taxa, given the broad taxonomic range represented. Moreover, it was hypothesized that both lipid class and FA composition would vary along the bathymetric gradient covered (~1000 m), with higher levels of unsaturation occurring at greater depths to compensate for pressure/temperature variations.

## Materials and methods

### Sampling

Organisms belonging to various taxa were opportunistically collected within 7 days in November-December 2013, during one of the annual multispecies bottom-trawl surveys conducted by Fisheries and Oceans (DFO), Canada, onboard the CCGS *Teleost*. Sampling followed a stratified random design with a minimum of two sets per stratum, and tow durations of ~ 15 min (~ 4.8 km h^−1^ gear opened and closed at sampling depth). Individuals were collected from a total of 23 tows inside a 100 km radius with a mean depth range of 313 to 1407 m. The gear used included a 16.9 m wide net with four panels of polyethylene twine. Further details are found in Walsh and McCallum [42]. Mean bottom temperature at the sampling site was 4.0 ± 0.3°C, with a slight decrease with depth. The sampling area, referred to as NAFO Division 3K, is located off Newfoundland, eastern Canada, in the Northwest Atlantic (49° 31’- 51° 51’N, 49° 32’- 51° 13’W). Once on board, individuals were immediately vacuum packed and frozen at -20°C to minimize lipid oxidation and hydrolysis. Individuals were identified to the lowest possible taxonomic level from direct observation and through photo-identification. A total of 284 deep-sea organisms, belonging to 139 species and 8 phyla, were weighed for total wet mass (post-freezing), once in the lab, and processed for lipid analysis at the CREAIT-ARC Facility of Memorial University (Table 1). Tissues characterized by low turnover rates were purposely selected for analysis, since they provide longer-term information. Specifically, the following tissues were sampled, as recommended by previous investigators [43]: dorsal white muscle from fish; body wall and tube feet from echinoderms; foot muscle from gastropods; mantle from cephalopods; non-gonad soft tissues or body walls from cnidarians; and dorsal abdominal muscle from crustaceans. When collection of target tissues was not feasible due to small body size, whole individuals were processed after being rinsed with distilled water. This was the case for 5 individuals of the phylum Annelida (i.e. *Alitta succinea*, Nereididae sp. 1, Polychaeta sp. 1, Polynoidae sp. 3, and Prionospio sp.), 10 of the Arthropoda (species of *Arcoscalpellum michelottianum* and *Nymphon* spp.), 2 of the Chordata (i.e. Ascidiacea sp. 3, and *Eudistoma vitreum*), and 3 of the Echinodermata (species of Gorgonocephalus sp., and *Ophioscolex glacialis*).

**Table 1:** Deep-sea macrofauna analyzed. Phylum, class, and species analyzed, together with sample size, mean depth of collection, and mean values ±sd of wet mass and total lipid content are shown. Data are reported from the phylum containing the highest amounts of lipids to the phylum characterized by the lowest contents.

Phylum	Class	Species	N	Depth	Mean wet mass	Mean total lipid content
				m	g±sd	mg g^-1^ wm±sd
**Chordata**	** **	* *				
** **	Actinopterygii					
		*Alepocephalus bairdii*	2	707–1321	161.2±140.0	41.0±31.2
		*Anoplogaster cornuta*	4	919–1365	80.2±26.9	148.0±30.6
		*Antimora rostrata*	3	1090	263.2±92.3	2.9±0.3
		*Arctozenus risso*	2	1090	15.1±15.6	67.3±75.2
		*Bathylagus euryops*	2	1090	7.5±2.4	19.7±11.7
		*Bathytroctes macrolepis*	2	1282	67.3±35.2	6.0±2.1
		*Borostomias antarcticus*	4	1090–1321	44.9±46.7	22.1±17.5
		*Caristius macropus*	1	1365	176.3	172.4
		*Chauliodus sloani*	6*	889–1365	36.7±12.3	25.9±15.9
		*Chiasmodon niger*	3	1365	78.9±45.4	568.9±417.4
		*Coryphaenoides rupestris*	3	759	54.3±23.4	4.8±1.9
		*Cottunculus microps*	2	889–919	72.4±12.2	7.7±8.3
		*Cottunculus thomsonii*	1	1090	1379.3	34.2
		*Cyclothone microdon*	2	1090	6.6±0.1	26.4±11.2
		*Gaidropsarus ensis*	4	919–1090	174.6±139.1	2.1±1.1
		*Glyptocephalus cynoglossus*	3	488	321.5	4.8±2.1
		*Haplophryne mollis*	1	1084	19.0	2.4
		*Lampadena speculigera*	1	1090	12.4	90.5
		*Lampanyctus* spp.	4	1090	24.9±6.5	52.2±32.2
		*Lepidion eques*	1	868	130.6	4.7
		*Macrourus berglax*	5*	759–1090	90.6±39.0	4.4±0.7
		*Magnisudis atlantica*	2	1122–1321	342.5±28.8	61.9±8.3
		*Malacosteus niger*	2	313–1094	38.8±0.9	68.0±2.9
		*Melanocetus johnsonii*	1	1407	178.8	7.4
		*Myctophum* sp.	1	1090	6.2	215.4
		*Nezumia bairdii*	3*	1090	97.2±54.5	4.7±2.8
		*Notacanthus chemnitzii *	3*	-	691.3±84.4	12.8±7.3
		*Notoscopelus* spp.	2	1090	21.7±6.8	270.1±9.2
		*Oneirodes macrosteus*	1	759	119.0	6.3
		*Polyacanthonotus rissoanus*	3*	1090–1321	94.2±34.6	33.0±17.6
		*Reinhardtius hippoglossoides*	2*	759–1090	542.9	141.7±148.2
		*Scopeloberyx opisthopterus*	2	1090	3.8±0.2	22.8±2.8
		*Scopelosaurus lepidus*	1*	759	128.8	43.6
		*Sebastes mentella*	3	488	200.0±108.1	13.8±2.2
		*Serrivomer beanii *	3	-	49.5±20.8	11.7±2.8
		*Synaphobranchus kaupii*	3	1090	100.0±14.7	156.6±99
		*Trachyrincus murrayi*	3	868	94.2±5.4	2.8±0.4
		*Xenodermichthys copei*	4	759–889	18.6±4.8	28.2±11
	Ascidiacea	* *				
		Ascidiacea sp 1	4**	759–1407	69.0±80.5	0.8±0.6
		Ascidiacea sp 2	1*	759	4.1	0.3
		Ascidiacea sp 3	1*	313	0.9	1.4
		Ascidiacea sp 4	2	759	7.4±0.6	3.9±0.9
		*Didemnum* sp.	1	759	0.9	1.9
		*Eudistoma vitreum*	1	1122	0.9	3.1
	Chondrichthyes					
		*Amblyraja jenseni*	1	919	796.7	8.1
		*Apristurus profundorum*	3	1324–1365	1805.4±249.5	9.0±3.1
		*Centroscyllium fabricii*	2	919	1177.7±72.8	6.8±0.5
		*Malacoraja senta*	1	759	81.7	11.1
		*Rajella fyllae*	4	919–1365	4.5±0.9	12.0±3.9
**Arthropoda**	** **	* *				
** **	Hexanauplia	* *				
		*Arcoscalpellum michelottianum*	3	1094–1365	6.6±1.5	10.6±5.1
	Malacostraca					
		*Acanthephyra pelagica*	3*	1090	7.0±0.9	34.0±7.7
		Anonyx sp 1	1	1365	0.6	94.6
		Anonyx sp 2	1	1321	0.7	281.6
		*Gnathophausia zoea*	3*	1090–1282	1.5±0.6	20.5±2.3
		*Munida tenuimana*	1	868	1.1	1.3
		*Munidopsis curvirostra*	3	1084–1282	1.6±0.4	33.5±42.7
		*Notostomus robustus*	1	1365	11.9	5.2
		*Pandalus borealis*	3	488	5.6±0.2	15.8±7.5
		*Pasiphaea tarda*	3	1321	29.2±15.5	8.7±5.4
		*Sabinea hystrix*	3	1090–1094	7.0±3.2	11.6±3.1
		*Stereomastis sculpta*	3	1094–1321	4.6±2.1	4.4±2.3
		*Themisto libellula*	1	313	0.1	8.5
	Pycnogonida	* *				
		*Nymphon* spp.	6*	347–868	0.3±0.2	8.7±6
**Echinodermata**		* *				
** **	Asteroidea	* *				
		*Astropecten americanus*	3	1122	14.3±3.02	18.2±13.0
		*Brisingida* spp.	2	1084–1365	52.6±41.9	24.1±16.8
		*Cheiraster* sp.	1	1365	3.5	0.4
		*Ctenodiscus crispatus*	3	313	3.1±1.0	2.6±0.4
		*Freyella microspina*	1	1407	70.2	103.8
		*Leptychaster arcticus*	3	353	2.4±0.3	5.9±1.3
		*Mediaster bairdi bairdi*	3	1090	14.8±3.5	4.2±0.2
		*Myxaster sol*	1	919	71.1	5.7
		*Psilaster andromeda*	2*	868–1365	19.6±19.1	31.5±42.7
		*Zoroaster fulgens*	3	759–1282	16.8±17.8	16.6±26.6
	Echinoidea	* *				
		*Brisaster fragilis*	2*	759	3.7±2.5	1.9±2.6
		*Phormosoma placenta*	3	889	19.6±7.4	6.0±2.7
		*Strongylocentrotus pallidus*	2	353–379	20.5±22.1	2.6±0.9
	Ophiuroidea					
		*Gorgonocephalus* sp.	1	595	1.2	42.4
		*Ophiopholis aculeata*	2	353	0.9±0.5	17.3±13.3
		*Ophioscolex glacialis*	2	353	0.7±0.3	15.3±2.6
		*Ophiura sarsii*	3	1282	6.7±1.4	1.5±0.6
**Annelida**	** **	** **	** **	** **	** **	
** **	Polychaeta	** **	** **	** **	** **	
		*Alitta succinea*	1	1027	0.3	16.8
		*Laetmonice filicornis*	1	595	3.1	7.4
		Nereididae sp 1	1*	868	0.0	8.5
		Nereididae sp 2	1	347	1.6	17.5
		Polynoidae sp 1	1	347	1.9	6.3
		Polynoidae sp 2	2	595	4±0.3	5.3±0.3
		Polynoidae sp 3	1	595	0.7	15.6
		Polychaeta sp 1	1	595	0.7	11.5
		*Prionospio* sp.	1	868	0.1	4.7
**Cnidaria**	** **	* *				
** **	Anthozoa	* *				
		*Acanella arbuscula*	3*	759–1122	5.5±3.6	3.3±0.2
		*Actinauge cristata*	2*	759–889	101.9±44.5	0.8±0.4
		*Actinoscyphia aurelia*	3**	796–1027	33.9±21.1	0.4±0.2
		*Actinostola callosa*	3**	759	71.4±26.7	0.3±0.1
		*Anthomastus agaricus*	3	1027	12.2±7.1	4.1±1.9
		*Anthomastus* sp.	1	868	5.2	5.4
		*Anthoptilum grandiflorum*	1	759	4.8	35.4
		*Duva florida*	1	-	15.8	14.5
		*Flabellum alabastrum*	2	759	6.5±2.3	11.7±0.4
		*Funiculina* sp.	1	1084	2.1	13.1
		*Paragorgia arborea*	1	595	90.3	13.3
		*Pennatula aculeata*	3	1282	2.0±0.6	14.7±4.7
		*Pennatula grandis*	2	759–1282	4.2±2.2	18.7±8.2
		*Umbellula* sp.	1	1122	3.8	31.1
	Scyphozoa	* *				
		*Atolla wyvillei*	3*	1090	25.5±24.5	0.7±0.7
		*Periphylla periphylla*	4**	759–1282	58.9±94.2	1.8±0.8
		Scyphozoa sp.	1*	1090	59.7	0.6
**Mollusca**	** **	* *				
** **	Cephalopoda	* *				
		*Bathypolypus arcticus*	3	464–1321	19.2±14.1	7.4±1.0
		*Bathypolypus bairdii*	1	707	50.1	4.3
		Cephalopoda sp 1	1	1282	410.9	9.2
		Cephalopoda sp 2	1	1407	986.8±127.4	2.8±1.4
		*Chiroteuthis veranii*	1	1090	151.2	12.0
		*Illex coindetii*	3	1282	54.2±7.2	10.2±3.2
		*Neorossia caroli*	1	488	17.2	5.2
		*Rossia megaptera*	1	1407	36.7	4.5
		*Stauroteuthis syrtensis*	3	1090–1407	22.1	7.2
	Gastropoda	* *				
		*Arrhoges occidentalis *	1	1282	6.2	4.4
		*Buccinum* sp.	3	759	5.8±2.6	6.9±1.3
		*Colus* spp.	3	759–889	22±30.3	4.8±1.0
		*Neptunea despecta*	1	889	7.1	4.8
**Porifera**	** **	* *				
** **	Demospongiae					
		*Cliona* sp.	1	1027	76.0	6.1
		*Craniella cranium*	3	464–595	13.1±6.1	6.9±1.0
		*Geodia* sp.	1	1027	577.9	5.1
		*Haliclona* sp.	2	1324	14.8±0.4	3.9±1.6
		*Hamacantha* (*Vomerula*) *carteri*	1	488	44.7	0.8
		*Histodermella* sp.	1	-	3.1	13.3
		*Iophon piceum*	1	353	157.2	7.8
		*Mycale (Mycale) lingua*	1	759	55.4	4.1
		*Phakellia* sp.	1	313	93.3	5.2
		*Polymastia* spp.	2	353	19.7±14.3	9.9±0.7
		*Polymastia hemisphaerica*	1	488	29.7	4.5
		*Stelletta* sp.	1	1122	26.1	4.3
		*Stryphnus ponderosus*	1	-	14.8	10.6
		*Tentorium semisuberites*	1	353	6.1	13.8
		*Thenea muricata*	4	353	16.2	2.6±1.2
	Hexactinellida					
		*Euplectella* sp.	2	1407–1094	87.7±107.4	4.3±3.7
		Hexactinellida sp 1	1	1027	228.6	4.8
		Hexactinellida sp 2	1*	1407	21.9	0.3
**Sipuncula**	** **	* *				
** **	Sipunculidea	* *				
		Sipunculidea sp 1	1	1407	3.5	7.3
		Sipunculidea sp 2	1	1122	2.0	3.0

*^,^ ** n = 1, 2 individual(s) removed from analysis of lipid composition

### Lipid extraction

An aliquot of tissue (0.7 ± 0.2 g) was sampled from each still-frozen individual to limit lipid oxidation and hydrolysis. Prior to lipid extraction, each sample was immersed in chloroform (4 or 8 ml, depending on tissue amount), sealed under nitrogen gas, and stored in a freezer (-20ºC). Lipids were extracted and analyzed based on Parrish [44]. Briefly, samples were homogenized in a chloroform:methanol:water (2:1:1) mixture, sonicated, and centrifuged four times. Lipid extracts were pooled in a lipid-clean vial following each wash, and the total amount was concentrated down to volume under a gentle stream of nitrogen. Vials were sealed and stored at -20ºC until further analysis.

### Total lipid content and lipid classes

Lipid extracts were analyzed using the Chromarod-Iatroscan TLC/FID system [45]. In detail, the lipid extracts were spotted on silica-gel coated rods (Chromarods-SIII) and developed in three solutions of different polarity, to allow lipid class separation. Samples were first developed in a mixture of hexane:diethyl ether:formic acid (98.95:1:0.05), which allowed the separation of hydrocarbons (HC), wax esters/steryl esters (WE/SE), ethyl esters (EE), methyl esters (ME), as well as ethyl and methyl ketones (EK and MK, respectively). Wax esters and steryl esters were considered together in this study as WE/SE, since the method used does not allow the separation of the two lipid classes. The second development, consisting of hexane, diethyl ether, and formic acid 79.9:20:0.1 led to the separation of diacyl glyceryl ethers (GE), triacyglycerols (TAG), free fatty acids (FFA), alcohols (AL), sterols (ST), and diacylglycerols (DAG). Lastly, acetone-mobile polar lipids (AMPL) and phospholipids (PL), the most polar among the lipid classes, were separated by the third development of 100% acetone followed by chloroform:methanol:chloroform-extracted-water (5:4:1). After each development, lipid classes were scanned on the rods using an Iatroscan MK V and quantified by combustion in a flame ionization detector. Lipid classes were identified and quantified through comparison with known standards, such as n-nonadecane for hydrocarbons, cholesteryl palmitate for SE, 3-hexadecanone for ketones, tripalmitin for triacyglycerols, palmitic acid for FFA, 1-hexadecanol for alcohols, cholesterol for sterols, 1-monopalmitoyl-rac-glycerol for acetone-mobile polar lipids, and DL-α-phosphatidylcholine dipalmitoyl for phospholipids. The sum of the amount of all the lipid classes in each sample provided the total lipid content (mg g^-1^ wet mass), while each lipid class was measured as percent of total lipids. Proportions of lipid classes were then used to calculate the triacylglycerol to sterol ratio (TAG:ST), or condition index [26], and the phospholipid to sterol ratio (PL:ST) as a measure of membrane fluidity [46, 47].

### FA analysis

FA were derivatized at 100ºC with H_2_SO_4_ in methanol, and quantified as methyl esters by gas chromatography. Briefly, an aliquot of the lipid extract, calculated in relation to the total amount of lipids within each tissue sample, was transferred into a lipid clean vial and evaporated under N_2_, to dryness. After adding 1.5 ml of dichloromethane and 3 ml of Hilditch reagent (i.e. H_2_SO_4_ dissolved in methanol) to samples, vials were sonicated, sealed, and heated for 1 hour at 100ºC. On cooling, 0.5 ml of saturated sodium bicarbonate and 1.5 ml of hexane were added to the solution, thus creating two layers. The upper, organic layer was removed and transferred into a new lipid-clean vial. Finally, the solution was blown dry under N_2_, and hexane (0.5 ml) was added to each vial. Samples were then sealed and loaded into a HP 6890 GC-FID equipped with a 7683 autosampler, for FA identification and quantification. Briefly, the column temperature was initially set at 65°C and held for 0.5 min. The temperature was raised to 195°C at a rate of 40°C min^-1^, held for 15 min, and then to a final temperature of 220°C at a rate of 2°C min^-1^, held for 0.75 min. Hydrogen was the carrier gas, which flowed at a rate of 2 ml min^-1^. The injector temperature started at 150°C and then raised to a final temperature of 250°C, at a rate of 120°C min^-1^. The detector temperature remained constant at 260°C. Peaks were identified comparing retention times from standards purchased from Supelco, including 37 component FAME mix (Product number 47885-U), Bacterial acid methyl ester mix (47080-U), PUFA 1 (product 47033) and PUFA 3 (47085-U). In this study, FA were reported as sums, whereas individual proportions may be found in Parzanini (unpublished; S4 Table). In detail, the sum of the saturated (∑Sat) was measured by summing the proportions of the following FA: 14:0, trimethyltridecanoic acid, 15:0, pristanic acid, 16:0, phytanic acid, 17:0, 18:0, 19:0, 20:0, 21:0, 22:0, 23:0, and 24:0. The sum of the monounsaturated FA (∑MUFA) was obtained by summing 14:1, 15:1, 16:1n-11, 16:1n-9, 16:1n-7, 16:1n-5, 17:1, 18:1n-11, 18:1n-9, 18:1n-7, 18:1n-6, 18:1n-5, 20:1n-11(13), 20:1n-9, 20:1n-7, 22:1n-11(13), 22:1n-9, 22:1n-7, and 24:1; whereas the polyunsaturated 16:2n-4, 16:3n-3?, 16:4n-3?, 16:4n-1, 18:2a, 18:2b, 18:2n-6, 18:2n-4, 18:3n-6, 18:3n-4, 18:3n-3, 18:4n-3, 18:4n-1?, 18:5n-3, 20:2α?, 20:2β?, 20:2n-6, 20:3n-6, 20:4n-6, 20:3n-3, 20:4n-3, 20:5n-3, 21:5n-3?, 22:4n-6, 22:5n-6, 22:4n-3?, 22:5n-3, 22:6n-3, and the non-methylene-interrupted-dienoic 22:2 (i.e. 22:2NIMDa?, 22:2NIMDb?) were summed to calculate ∑PUFA. For the sum of the n-3 and n-6 FA, only those acids involved in the desaturation/elongation pathway were used, including 18:3n-3, 18:4n-3, 20:4n-3, 20:5n-3, 22:5n-3 and 22:6n-3 for ∑n-3, and 18:2n-6 18:3n-6, 20:3n-6, 20:4n-6, 22:4n-6 and 22:5n-6 for ∑n-6. Lastly, DHA+EPA represents the sum of the amounts of docosahexaenoic acid (22:6n-3) and eicosapentaenoic acid (20:5n-3) reported in g per 100-g of wet mass.

### Statistical analysis

Two types of mean values were reported in Results and Tables: i) averages per phylum ± se and ii) averages per species ± sd; and phyla are listed in decreasing order of mean lipid contents in the Results as well as in the Tables. To study the relative magnitude of data variability among and within phyla, the coefficient of variation (CV) was calculated for selected metrics (i.e. wet mass, total lipid content, and proportions of PL, FFA, ST, TAG, WE/SE). Due to analytical artifacts related to blank correction and the consequent underestimation of the proportion of PL in individuals with low lipid content, n = 28 samples were removed from all the analyses involving lipid class composition (Table 1). Based on non-normal data distributions and heterogeneity of variances, Spearman rank correlations were run to test for the presence of any relationship among depth of collection (mean value for each depth strata), total lipid content, lipid classes (PL, FFA, ST, TAG, and WE/SE), lipid ratios (TAG:ST and PL:ST), fatty acid indices (∑Sat, ∑MUFA, ∑PUFA, ∑n-3, ∑n-6), and wet mass of whole individuals. Furthermore, PERMANOVA (permutational multivariate ANOVA) and PCO (principal coordinate analysis) were performed to explore differences in lipid and FA composition across taxa. Specifically, a 1-factor PERMANOVA was initially run to test which factor, among “Phylum”, “Class”, or “Species” better explained the variability across organisms in terms of lipid class and FA composition. As “Phylum” was the best descriptor, a 2-factor PERMANOVA was subsequently performed to assess whether and to what extent “Depth”, in addition to “Phylum”, influenced the variability. Univariate analyses were run using the software Sigmaplot 11.0, and multivariate statistics was conducted in Primer 6 + PERMANOVA [48].

### Ethical approval

Field collections were performed by the Canadian Government's Fisheries and Oceans under their rules, regulations and permits.

## Results

### Lipid and FA composition across phyla

Lipid analysis was performed on deep-sea organisms across a wide range of taxa, body masses and depths (Table 1), and inside a tight temporal and geographical window. Representatives of the phyla Chordata and Arthropoda exhibited the highest mean concentrations of total lipids in their tissues, with marked variability (± se: Table 2). In particular, the Chordata displayed both the greatest lipid amounts (56.0 ± 12.1 mg g^-1^ wm, n = 105) and highest CV (221%), followed by Arthropoda (24.8 ± 9.0 mg g^-1^ wm, n = 32; 206%). Conversely, the Porifera (5.9 ± 0.7 mg g^-1^ wm, n = 25) and the Sipuncula (5.1 ± 2.2 mg g^-1^ wm, n = 2) contained the lowest lipid quantities. Lipid contents of all remaining taxa along with CVs are listed in Table 2.

**Table 2:** Wet mass and lipid profiles in deep-sea macrofauna phyla under study. Sample number (n), and mean values of wet mass, total lipids, and mean proportion of phospholipids (PL), free fatty acids (FFA), sterols (ST), triacylglycerols (TAG), wax esters or steryl esters (WE/SE). Coefficients of variation (CV; %) are also reported for each mean value, as well as grand means related to each variable.

Phylum	n	Wet mass		Total lipids		PL		FFA		ST		TAG		WE/SE	
		g±se	CV	mg g^-1^ wm±se	CV	%±se	CV	%±se	CV		%±se	CV		%±se		CV	%±se	CV
**Chordata**	105	186.0±36.7	202	56.0±12.1	221	24.7±2.1	85	20.5±1.6	79	11.0±0.9	84	24.9±2.7	113	3.7±1.1	311
**Arthropoda**	32	6.2±1.6	146	24.8±9.0	206	31.7±3.8	68	25.1±2.6	59	15.2±1.6	58	7.3±2.3	180	8.8±3.1	199
**Echinodermata**	35	16.2±3.5	129	14.3±3.6	151	45.6±3.4	44	14.7±1.5	61	14.3±1.4	58	7.1±1.9	155	0.1±0.0	297
**Annelida**	9	1.8±0.5	85	10.1±1.8	53	38.2±6.3	49	21.6±4.7	65	21.5±4.9	68	6.8±2.8	123	3.5±2.3	193
**Cnidaria**	25	16.9±5.2	154	9.8±1.9	98	28.5±3.1	54	20.1±2.4	59	12.2±0.9	37	5.4±1.4	128	12.7±1.8	71
**Mollusca**	23	172.4±70.0	195	6.4±0.6	44	66.4±2.8	20	15.0±1.9	59	16.9±1.1	31	0.4±0.3	288	-	
**Porifera**	25	73.3±25.6	174	5.9±0.7	59	45.6±3.7	41	17.6±1.6	45	17.9±1.2	35	5.3±1.1	107	3.2±1.2	181
**Sipuncula**	2	2.8±0.8	39	5.1±2.2	59	52.8±16.4	44	5.1±2.8	79	35.9±14.8	58	-	-	
**Mean CV**			**141**		**111**		**51**		**63**		**54**		**156**		**209**

**Fig 1:**
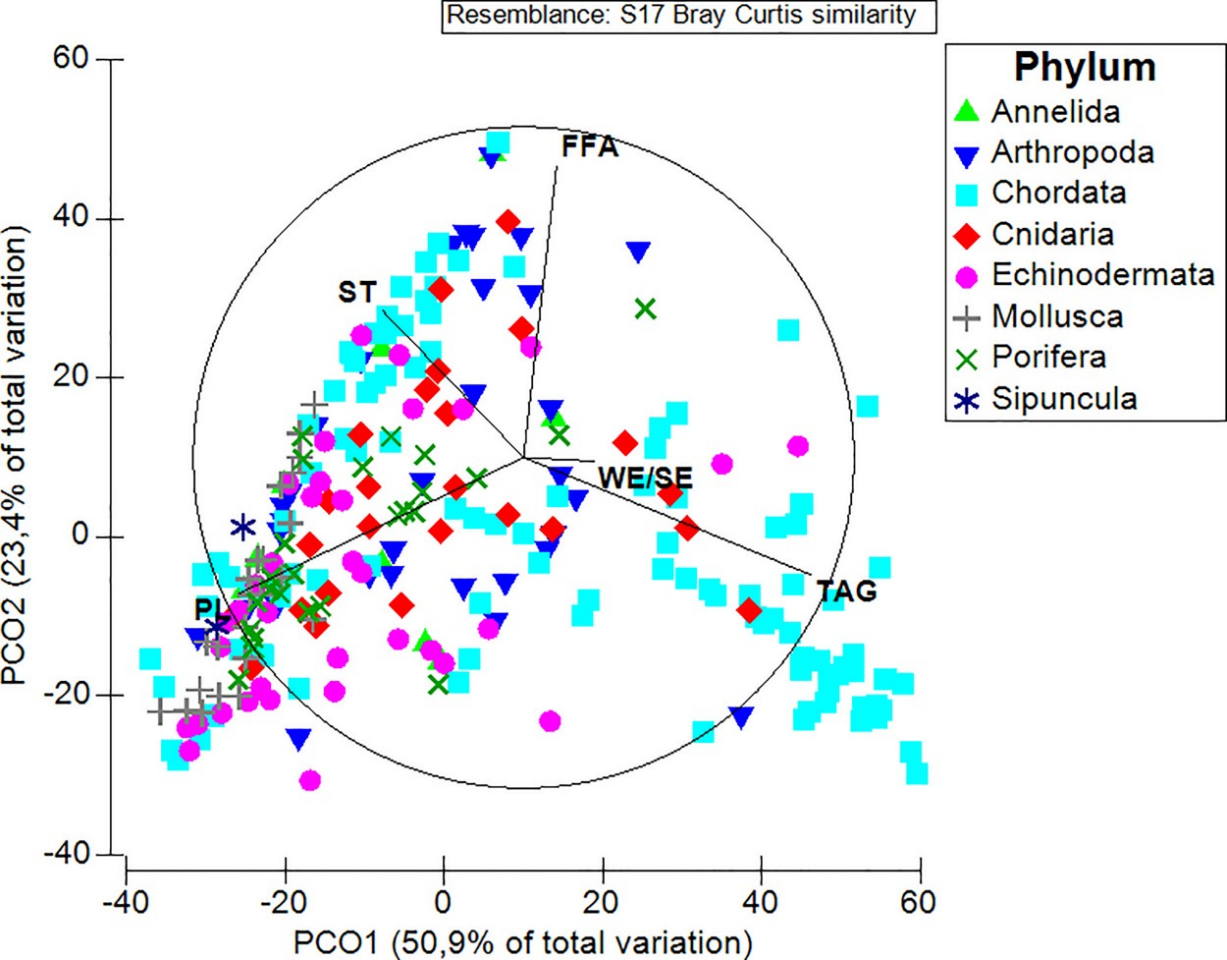
Principal coordinate (PCO) analysis plot representing differences in terms of lipid class composition across phyla. The lipid classes reported occurred with proportions > 1.7%, including phospholipids (PL), free fatty acids (FFA), sterols (ST), triacylglycerols (TAG), and wax esters/steryl esters (WE/SE).

A total of 14 lipid classes were represented within the faunal assemblage. Overall, PL (35.3 ± 1.5%), FFA (19.4 ± 0.9%), ST (13.9 ± 0.6%), TAG (13.4 ± 1.3%), and WE/SE (4.3 ± 0.7%) were the most abundant lipid classes across all individuals analyzed (n = 256). The remaining lipid classes (i.e. HC, EE, ME, EK, MK, GE, AL, DAG, and AMPL) occurred in smaller mean proportions (< 1.7%) and, for this reason, they were not further considered in the analysis; nonetheless, their proportions within each phylum is reported in S1 Table of the Supplementary Material. PL dominated the lipid class composition of all phyla analyzed, with mean proportions ranging from 24.7 ± 2.1% in the Chordata to 66.4 ± 2.8% in the Mollusca (Table 2). FFA and ST were similarly detected in all the phyla, although to a generally lower extent than PL, ranging from 5.1 ± 2.8% in the Sipuncula to 25.1 ± 2.6% in the Arthropoda, for the former, and from 11.0 ± 0.9% in the Chordata to 35.9 ± 14.8% in the Sipuncula, for the latter (Table 2). While the Chordata had high levels of TAG in their tissues, i.e. 24.9 ± 2.7, this lipid class was less abundant in the other phyla (< 8%), and it was absent in the Sipuncula (Table 2). WE/SE were detected in all phyla except for the Mollusca and Sipuncula, with the Arthropoda and Cnidaria having the highest mean proportions (8.8 ± 3.1 and 12.7 ± 1.8%, respectively; Table 2). Overall, the lipid class composition varied significantly among phyla and depths at collection (PERMANOVA, *Pseudo-F*_*7*, *244*_ = 4.8, p(perm) = 0.0001, with “Phylum” as factor; *Pseudo-F*_*48*, *244*_ = 1.4, p(perm) = 0.0031, with “Depth” as factor; and *Pseudo-F*_*48*, *244*_ = 1.4, p(perm) = 0.0031, “Depth X Phylum” as factor). PL and TAG influenced PCO1, which accounted for 50.9% of the variation among samples (Fig 1). In addition, the mean CV measured for TAG and WE/SE was higher (> 150%) than that measured for PL, FFA, and ST (Table 2). Regarding the lipid ratios, the condition index TAG:ST ranged from values close to 0 in the Mollusca, Porifera, and Annelida, to 7.7 ± 1.6 in the Chordata. Despite the low values of the index, Mollusca also displayed the highest CV (Table 3). Conversely, results for the PL:ST were less variable across taxa overall, and values ranged from 1.8 ± 0.4 in the Annelida to 4.5 ± 0.5 in the Mollusca (Table 3).

**Table 3:** Lipid class ratios across phyla. Mean values ±se of triacyglycerols to sterols (TAG:ST) ratio and phospholipids to sterols (PL:ST) ratio reported for each phylum, together with corresponding coefficients of variation (CV; %).

Phylum	TAG:ST		PL:ST	
	Mean±se	CV	Mean±se	CV
**Chordata**	7.7±1.6	203	3.2±0.5	147
**Arthropoda**	1.3±0.6	250	3.7±0.8	127
**Echinodermata**	0.9±0.3	173	4.0±0.5	72
**Annelida**	0.5±0.2	128	1.8±0.4	66
**Cnidaria**	0.6±0.2	141	2.5±0.3	53
**Mollusca**	0.0±0.0	325	4.5±0.5	49
**Porifera**	0.3±0.1	155	3.1±0.4	62
**Sipuncula**	-	-	2.0±1.3	91
**Mean CV**		**196**		**83**

**Fig 2:**
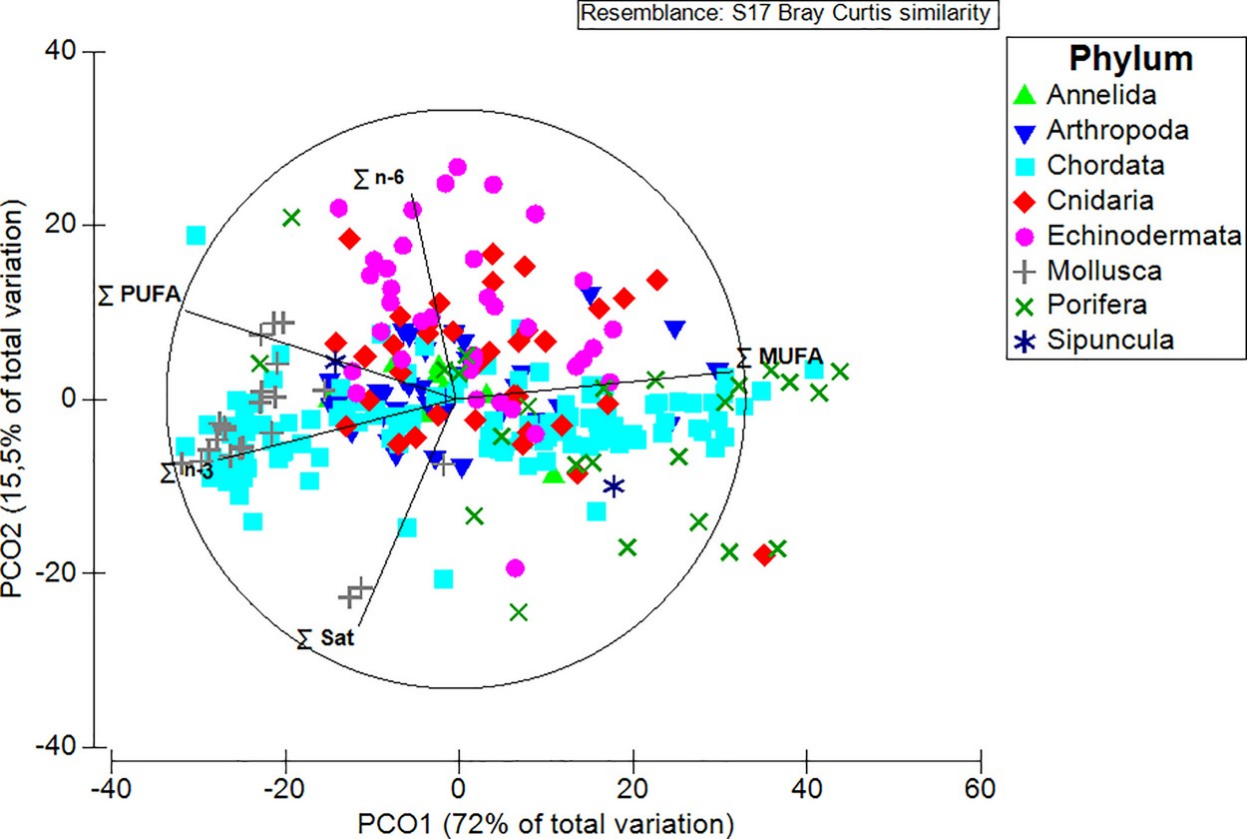
Principal coordinate (PCO) analysis plot representing differences in terms of FA composition across phyla. The sums of saturated- (∑ Sat), monounsaturated- (∑ MUFA), and polyunsaturated FA (∑ PUFA), are reported together with the sums of n-3 and n-6 FA (∑ n-3 and ∑ n-6, respectively).

Mean proportions (±se) of saturated FA (∑Sat) ranged from 14.9 ± 1.3% in the Echinodermata to 26.9 ± 2.1% in the Mollusca, and unsaturated FA (∑MUFA and ∑PUFA) were generally higher than saturated FA in all phyla, except Mollusca (Table 4). In fact, this phylum was characterized by lower mean proportions of ∑MUFA than those of ∑Sat and ∑PUFA, as shown in Table 4. Regarding the essential FA, mean levels of ∑n-3 were higher overall (from 11.7 ± 2.0% in the Porifera to 42.4 ± 2.8% in the Mollusca) than those of ∑n-6 (from 2.1 ± 0.5% in the Porifera to 14.4 ± 1.8% in the Echinodermata). Overall, the FA composition was significantly different across phyla (PERMANOVA, *Pseudo-F*_*7*, *278*_ = 9.6, p(perm) = 0.0001, with “Phylum” as factor; *Pseudo-F*_*24*, *278*_ = 1.2, p(perm) = 0.1614, with “Depth” as factor; and *Pseudo-F*_*51*, *278*_ = 1.2, p(perm) = 0.1782, “Depth X Phylum” as factor), and ∑MUFA and ∑PUFA influenced PCO1, which accounted for 72.0% of the variation among samples (Fig 2). In particular, pairwise comparisons indicated that both the Annelida, Arthropoda, and Chordata were significantly different from the Echinodermata, Mollusca, and Porifera (p(perm) < 0.05); the Cnidaria and Echinodermata were significantly different from the Mollusca and Porifera instead (p(perm) = 0.0001); and, lastly, the Mollusca significantly differed from the Porifera and Sipuncula (p(perm) < 0.05). Representatives of the phylum Chordata presented the highest mean concentrations of DHA+EPA in their tissues (0.5 ± 0.1 g per 100-g wm), followed by those belonging to the phyla Arthropoda, Mollusca, and Echinodermata (0.2 ± 0.0, 0.2 ± 0.0, 0.2 ± 0.1 g per 100-g wm, respectively; Table 4). In general, the average CV measured for all the FA indices was <50%, with the only exceptions being those calculated for ∑n-6 and DHA+EPA, which were ≥85% (Table 4).

**Table 4:** Fatty acid sums characterizing the phyla under study. Sample number (n), mean value ±se and related coefficient of variation (CV; %) of the sum of saturated (∑Sat), monounsaturated (∑MUFA), polyunsaturated (∑PUFA), n-3 and n-6 FA, as well as DHA+EPA are reported for each phylum.

Phylum	n	∑Sat		∑MUFA		∑PUFA		∑n-3		∑n-6		DHA+EPA	
		%±se	CV	%±se	CV	%±se	CV	%±se	CV	%±se	CV	g per 100 g wm±se	CV
**Chordata**	115	22.4±0.7	34	42.0±1.7	44	33.9±1.3	42	27.7±1.3	49	3.7±0.3	76	0.5±0.1	179
**Arthropoda**	35	16.5±1.2	44	43.8±1.6	22	37.3±1.3	21	30.4±1.7	32	3.5±0.6	106	0.2±0.0	81
**Echinodermata**	36	14.9±1.3	52	43.1±1.5	21	40.3±1.6	24	18.6±1.4	46	14.4±1.8	74	0.2±0.1	149
**Annelida**	9	20.4±1.3	20	38.8±2.1	16	39.5±2.6	20	27.6±2.7	29	4.6±0.6	39	0.1±0.0	59
**Cnidaria**	35	17.6±0.9	30	44.4±1.6	21	35.4±1.6	27	21.4±1.6	44	10.0±1.5	91	0.1±0.0	166
**Mollusca**	23	26.9±2.1	37	19.3±1.0	25	53.2±2.1	19	42.4±2.8	32	5.4±1.2	108	0.2±0.0	79
**Porifera**	24	20.8±2.2	51	50.3±3.2	31	20.8±3.7	86	11.7±2.0	85	2.1±0.5	115	0.04±0.0	118
**Sipuncula**	2	26.7±3.3	17	36.3±12.2	48	34.0±16.3	68	12.0±7.3	86	8.2±4.1	70	0.03±0.0	132
**Mean CV**		**36**		**29**		**38**		**50**		**85**	** **	**120**

While ST negatively correlated with total lipid contents (*r*_*s*_ = -0.6, n = 256, p = 0.000), both TAG and the TAG:ST ratio positively correlated with total lipid amounts (TAG, *r*_*s*_ = 0.6, n = 256, p = 0.000; TAG:ST, *r*_*s*_ = 0.7, n = 250, p = 0.000). Although no significant relationship was detected between total lipid content and wet mass, ST negatively correlated with wet mass (*r*_*s*_ = -0.2, n = 256, p = 0.004). Although weak, significant correlations were found between depth and various metrics. Specifically, depth correlated positively with total lipid content (*r*_*s*_ = 0.2, n = 256, p = 0.001); wet mass (*r*_*s*_ = 0.2, n = 256, p = 0.001); PL:ST (*r*_*s*_ = 0.1, n = 238, p = 0.026) and ∑MUFA (*r*_*s*_ = 0.2, n = 270, p = 0.002). In contrast, it correlated negatively with FFA (*r*_*s*_ = -0.2, n = 256, p = 0.009); ST (*r*_*s*_ = -0.2, n = 256, p = 0.000); and ∑n-6 (*r*_*s*_ = -0.2, n = 270, p = 0.003).

### Lipid and fatty acid composition within phyla

#### Chordata

Overall, representatives of this phylum were characterized by the highest mean levels of lipids in their tissues, as well as the greatest mean proportions of TAG. Lipid data were highly variable across the taxa in the Chordata, with CV of mean values being ≥ 113% for both total lipid content and TAG (Table 2). Ray-finned fish (Actinopterygii) showed higher amounts of lipid in their tissues than sharks (Chondrichthyes) and tunicates (Ascidiacea), with values ranging from 2.1 ± 1.1 mg g^-1^ wm in *Gaidropsarus ensis*, to 569.0 ± 417.0 mg g^-1^ wm in *Chiasmodon niger* (Table 1). Ray-finned fish also had a different lipid class composition, with high proportions of TAG, up to 82.9 ± 6.2% in *C*. *niger* (S2 Table). In contrast, PL was the prevailing lipid class in the muscle tissue of sharks and ascidians, and with ST representing an important fraction in the body wall of the latter (≥ 23.7 ± 9.5%; S2 Table). Although the phylum was characterized overall by low levels of WE/SE, the fish *Arctozenus risso*, *Borostomias antarcticus*, *Caristius macropus*, *Lampadena speculigera*, and *Lampanyctus* spp. presented proportions of this lipid classes > 17% (S2 Table). Conversely, variation in fatty acid data was smaller, and the Chordata showed similar proportions of most FA indices, except for ∑n-6 where CVs reached 76% (Table 4). In detail, mean values of ∑n-6 ranged from 1.3% in *Oneroides macrosteus* to 12.8 ± 2.1% in Ascidiacea sp. 4 (S3 Table). Tunicates were in general characterized by higher mean levels of n-6 FA in their tissues, whereas sharks had larger proportions of PUFA and n-3, and ray-finned fish of ∑Sat (S3 Table).

#### Arthropoda

Malacostraca crustaceans had higher levels of lipids in their tissues than Pycnogonida and Hexanauplia representatives (Table 1). Furthermore, most of lipids of these crustaceans was represented by WE/SE, as in *Acanthephyra pelagica*, *Anonyx* spp., and *Gnathophausia zoea* where this lipid class accounted for > 38% in (S2 Table). Conversely, the lipid profile of both Pycnogonida and Hexanauplia was mainly composed of PL and ST, with the former group also having high proportions of FFA (S2 Table). In addition, WE/SE was either absent or present at trace levels within Pycnogonida and Hexanauplia (≤ 0.2 ± 0.3%), whereas TAG occurred in higher mean proportions (≥ 11.2 ± 3.0%). Mean proportions of FA indices were similar overall within the phylum, with CV <45%, with the exception of ∑n-6 whose CV was 106% (Table 4). In detail, the two species in the genus *Anonyx* presented the lowest proportions of ∑n-6 (0.8 and 0.7%) *versus* 10.1 ± 11.3% in *Steromastis sculpta* (S3 Table). Overall, decapods, such as *S*. *sculpta*, *Pandalus borealis*, and *Notostomus robustus*, displayed the highest levels of ∑Sat and PUFA within the phylum.

#### Echinodermata

Echinoderms had relatively high amounts of lipids in their tissue (Table 2), dominated by PL (45.6 ± 3.4%). WE/SE were present only at trace levels in the sea star *Astropecten americanus*, the sea urchin *Strongylocentrotus pallidus*, and the brittle star *Ophiopholis aculeata*, whereas TAG was detected in most of the species, with particularly high mean proportions in the brittle stars *Ophiopholis aculeata* and *Ophioscolex glacialis* (S2 Table). While CVs of mean levels of ∑MUFA, ∑PUFA, and ∑n-3 was < 50% across echinoderms, greater variation was found for ∑Sat and ∑n-6 (Table 4). In fact, whereas proportions of ∑Sat ranged between 6.6% and 27.9%; levels of ∑n-6 ranged between 0.9 ± 0.9% and 29.5% (S3 Table).

#### Annelida

The Annelida had intermediate amounts of lipids (10.1 ± 1.8 mg g^-1^ wm), which were mostly represented by PL, FFA, and ST (Table 2); nonetheless, both TAG (6.8 ± 2.8%) and WE/SE (3.5 ± 2.3%) were also detected. In particular, Polynoidae sp 3 and *Alitta succinea* respectively had the highest proportions of TAG and WE/SE within the phylum. Proportions of saturated, unsaturated, n-3 and n-6 FA were similar overall across the Annelida. Mean levels of MUFA and PUFA were higher than those of ∑Sat, and proportions of ∑n-3 were larger than those of ∑n-6 (Table 4).

#### Cnidaria

In general, the Cnidaria had low amounts of lipids in their tissues, although results were variable (CV = 98%; Table 2). The highest total lipid contents were found in sea pens (class Anthozoa) such as *Anthoptilum grandiflorum* and *Umbellula* sp. (35.4 and 31.1 mg g^-1^ wm, respectively), whereas lipid levels in jellyfishes (Scyphozoa) were low at 2.0 ± 0.9 mg g^-1^ wm. Together with PL, FFA, and ST, WE/SE represented a significant fraction across cnidarians, with mean percentages of 12.7 ± 1.8% (Table 2), and the lipid class was particularly abundant in the corals *Paragorgia arborea* and *Umbellula* sp., as well as in the jellyfish *Periphyllia periphyllia* (S2 Table). Proportions of WE/SE were generally higher than those of TAG (Table 2). While proportions of ∑Sat, ∑MUFA, ∑PUFA and ∑n-3 were similar across the Cnidaria, marked variation was noted for ∑n-6, especially within the class Anthozoa (S3 Table). The sea anemone *Actinauge cristata* had the lowest levels of n-6 FA in its tissue (0.3 ± 0.1%), and the soft coral *Duva florida* had the largest proportions (40.4%).

#### Mollusca

The low mean value of lipid content was somewhat consistent across the Mollusca, with a CV of 44%. Likewise, the lipid class composition was similar among the species analyzed in this group with PL the most abundant lipid class, occurring with percentages > 53%. Furthermore, no WE/SE were detected and TAG levels were low and measured only in the body wall of the cephalopods *Illex coindetii* and *Neorossia caroli*, and in the gastropod *Arrhoges occidentalis* (S2 Table). Levels of ∑Sat, ∑MUFA, ∑PUFA, and ∑n-3 were similar across species, with CV < 40% and ∑n-6 showing the greatest variability (Table 4) from 0.5% in the cephalopod *Rossia megaptera* to 16.7 ± 5.4% in gastropods of the genus *Colus* (S2 Table).

#### Porifera

Sponges were characterized overall by a low lipid content (5.9 ± 0.7 mg g^-1^ wm), with PL representing the largest fraction (45.6 ± 3.7%). Most of the variability among species was detected in TAG and WE/SE, with TAG presenting higher mean proportions in demosponges, and WE/SE in glass sponges (S2 Table). Levels of PUFA, and n-3 and n-6 FA were highly variable across species (Table 4). In particular, the Hexactinellida had higher levels of PUFA than the Demospongiae, but the demosponge *Tentorium semisuberites* had the highest proportions of n-3 and n-6 FA in its tissue (30.7 and 6.8%, respectively).

#### Sipuncula

This phylum was represented by 2 species (Table 1). The Sipuncula had the lowest mean quantities of lipids among all the phyla analyzed (5.1±2.2 mg g^-1^ wm; Table 2), and most of these lipids were represented by PL, FFA, and ST; no TAG and WE/SE were detected in their tissues (Table 2). The 2 species of Sipuncula generally had higher mean levels of unsaturated FA, whereas those of ∑n-3 and ∑n-6 were similar (Table 4).

## Discussion

The present study explored a broad assemblage of 139 deep-sea species distributed across 8 phyla, which were collected within a tight spatial and temporal window along shelf and slope areas off Newfoundland, in the Northwest Atlantic. This sampling strategy was purposely adopted to minimize environmentally-driven variability in lipid content and composition, as well as to facilitate the comparative study of these parameters across taxa. Furthermore, tissues characterized by low turnover rates, and thought to incorporate longer-term data, were sampled from each taxon to reduce variability among tissue types and to optimize comparisons. When collection of these tissues was not feasible, due to the small size of certain taxa, entire organisms were processed instead to allow for lipid extraction [44]. While only a small proportion of individuals was analyzed as whole bodies (8%), comparisons involving representatives of the species *Alitta succinea*, Nereididae sp. 1, Polychaeta sp. 1, Polynoidae sp. 3, and *Prionospio* sp. (phylum Annelida); *Arcoscalpellum michelottianum* and *Nymphon* sp. (phylum Arthropoda); Ascidiacea sp. 3, and *Eudistoma vitreum* (phylum Chordata); and *Gorgonocephalus* sp. and *Ophioscolex glacialis* (Echinodermata) may have been less comparable with those from other taxa. As the phylum Sipuncula was represented by only 2 individuals, results for this taxon remain tentative. Nevertheless, they were still included in the analyses given the rarity and scientific value of deep-water samples.

As expected, there were marked differences in lipid content and composition both across the highest taxonomic groups (i.e. inter-phyla), as well as within phyla and within/among some of the lower taxonomic levels. Part of these differences may have been a reflection of phylogenetic diversity, as the PL composition of marine organisms is mostly driven by phylogeny [49] and PL represented the most abundant lipid fraction across the taxa analyzed. However, in the present study, most of the variability in lipid amounts appeared to be related to the lipid classes TAG and WE/SE (see paragraph below for assumptions and interpretations made for WE/SE), which also exhibited the largest coefficient of variation. In fact, both these lipid classes were positively correlated with total lipid content. As TAG and WE/SE are typical storage lipids in marine organisms [21, 26], such variability most likely reflects the different energy allocation strategies (i.e. how energy is distributed towards growth, survival, and reproduction) characterizing the taxa analyzed. Indeed, not all taxa accumulated energy reserves: the Mollusca and Sipuncula, whose lipid class composition was dominated by membrane lipids (i.e. PL+ST), had trace levels of storage lipids (TAG+WE/SE). Among those that did accumulate lipid stores, different lipid classes (e.g. TAG *vs* WE/SE) were used. For instance, whereas the Chordata and Echinodermata had relatively high proportions of TAG, the Cnidaria accumulated their energy storage in WE/SE instead. Lastly, representatives of the Arthropoda, Annelida, and Porifera used both TAG and WE/SE to store energy.

As previously shown by Lockyer [50], Fraser [26], and Lloret and Planes [51], lipid content and composition of organisms may fluctuate on broad scales according to foraging and storage modes, metabolism (e.g. low *vs* fast), reproductive strategies, environmental conditions, and food availability. Regarding the latter, studies suggest that high spatial and temporal variability in food supply selects for larger proportions of storage lipids [52]. At the intraspecific level, age, size, and sex may also play a role [26, 53]. Indeed, the size of organisms analyzed in the current investigation was highly variable within species, although no significant correlation was found overall between wet mass and lipid content and lipid class composition; whereas age and sex were not determined.

A positive correlation was detected, in the current study, between total lipid content and the condition index TAG:ST, suggesting that the fattier individuals were characterized by greater energy reserves than their conspecifics. This is mostly the case for the representatives of phylum Chordata, which had the highest variability in TAG:ST among and within species, as in *Notoscopelus* spp. and *Reinhardtius hippoglossoides*. In fact, as previously reported for shallow-water fish [51], corals [54], crustaceans [55], and bivalve larvae [26], the higher the lipid content and energy reserves within the representatives of these taxa, the higher their growth rate, reproductive success, or survival. The same idea may be applied to deep-sea organisms, taking into account that their metabolic rates and lipid stores are typically lower than in their shallow-water counterparts, and hence the way the energy is partitioned among somatic growth, reproduction, and survival may be different [52].

Certain crustaceans, fish, jellyfishes, and corals analyzed in this study used WE, rather than TAG, as the main form of energy storage. Although most terrestrial and aquatic organisms store energy in TAG [1], these crustaceans, fish, jellyfishes, and corals had greater levels of WE and/or SE, which could not be fully distinguished (S2 Table). Among them, the crustaceans *Acanthephyra pelagica*, *Anonyx* spp., and *Gnathophausia zoea*, the fish *Lampanyctus* spp., *Caristius macropus*, and *Arctozenus risso*, the jellyfishes *Atolla wyvellei* and *Periphylla peryphylla*, and the corals *Paragorgia arborea* and *Umbellula* sp. showed proportions of WE/SE >20% up to 60%. No indication was found in the literature about SE accumulation in these taxa. Whereas the technique applied in the current study did not allow for the separation of these two classes, Kayama et al. [56] found that proportions of SE were consistently smaller relative to those of WE in the roe of various shallow-water fish species, and Nevenzel [57] indicated that small amounts of SE are typically present in animal tissues. Therefore, the high proportion of WE/SE was assumed to mostly correspond to WE, which are known to play an important role as both energy storage and in buoyancy control [21, 25].

Deep-water zooplankton and fish were previously shown to accumulate large quantities of WE within their tissues [21, 58]. In particular, polar and sub-polar herbivorous zooplankton (e.g. copepods) accumulated large quantities of WE over summer, and used these lipids to store energy during long periods of starvation and to maintain neutral buoyancy at depths > 500 m [21, 59]. While TAG are used as a short-term deposit, WE provide a longer-term energy provision to such zooplankton overwintering at great depths [21]. Furthermore, the use of WE for buoyancy control is beneficial for zooplankton living in cold deep waters, due to the thermal expansion and compressibility of such molecules [59]. As for cold-water corals, the only study providing evidence of storage via WE is that conducted by Hamoutene et al. [19] within the same region of the Northwest Atlantic during the same season. Hamoutene et al. [19] proposed that corals stored their energy in WE, as well as in alkyldiacylglycerols. Here, the proportion of alkyldiacylglycerols (or glyceryl ethers) across all the Cnidaria species was minimal (0.05 ± 0.05%; S1 Table), hence not considered in the analysis. Conversely, they showed higher levels of TAG (S1 Table), suggesting these species may use both TAG and WE for energy storage as reported in shallow-water corals [19]. While herbivorous zooplankton are able to synthesize WE *de novo* [21], higher-level consumers can accumulate this lipid by incorporating it through diet [58]. It is likely that the crustaceans, jellyfishes, corals, and fish presenting larger levels of WE in the current investigation hence preyed on WE-rich zooplankton.

Depth was an important driver of lipid content and composition of the species analyzed in this study, and the environmental conditions at sampling might also have contributed to the variability in their lipid levels. Although sampling was carried out within a tight geographical radius (100 km), organisms were collected along a depth range of ~1000 m. Representatives of the phyla Mollusca and Echinodermata, for instance, which presented the highest PL:ST ratios, were collected between 464 and 1407 m and between 313 and 1407 m, respectively. According to Cossins and Macdonald [18] and Simonato et al. [60], environmental variables such as temperature and pressure may modulate lipid content and composition, and both these parameters vary along a bathymetric gradient [61]. Positive correlations were detected here between depth and the PL:ST ratio, an indicator of membrane lipid remodeling [1], as well as between depth and proportions of MUFA. However, depth negatively correlated with ST. These results suggest that both ST and unsaturated FA are involved in the bathymetric response and, specifically, that the species collected at deeper depths have overall higher levels of lipid unsaturation, mainly due to MUFA and a lower ST content. Decreasing temperature and increasing pressure along the depth gradient has the ability to reduce membrane fluidity, thus compromising its general structure and function [22, 46, 60]. In response, organisms may adjust and remodel the lipid composition of their membranes, through a process known as homeoviscous adaptation, which involves changes in the cholesterol content, as well as changes in length and unsaturation levels of the membrane FA and in phospholipid headgroups and molecular species [22, 60, 62]. Specifically, cholesterol, the main form of ST in most animals [16], generally favours packing in the membranes, increasing their rigidity [22]. In contrast, long-chain unsaturated FA are characterized by a higher molecular flexibility and lower melting points, thus providing more fluidity to membranes [63]. Direct evidence of this type of lipid remodelling was documented in shallow-water bivalves [47], as well as in deep-water microorganisms [64]. It was also suspected to occur in fish collected between 200 and 4000 m; specifically, deeper-water species were displayed higher levels of unsaturation than shallow-water ones [18].

Interestingly, included in the present dataset were species known to undergo diel vertical migration, such as the myctophid fish *Lampanyctus* spp. and *Myctophum* sp. [65] and the crustacean decapod *Acanthephyra pelagica* [66]. Since these species can travel vertically over a few hundred meters [66], thus experiencing marked changes in temperature and pressure, it would be of particular interest to undertake a study to assess their ability to overcome such variations in terms of membrane lipid composition. Pernet et al. [67] found that while the level of unsaturation was adjusted in response to both long- and short-term acclimation to temperature fluctuations in the shallow-water oyster *Crassostrea virginica*, the modulation of the PL:ST ratio was only accomplished in response to long-term acclimation. Hazel and Landrey [68] noted that the modulation of phospholipid molecular species and headgroups preceded the adjustment of the unsaturation level in the rapid thermal acclimation of the rainbow trout *Salmo gairdneri*, it would be valuable to verify whether deep-sea species have the same time course for thermal acclimation.

In the present study, FA composition was more consistent across phyla than the lipid class composition and this, probably, was mostly driven by phylogeny, in accordance with Dalsgaard et al. [69]. In addition, higher proportions of unsaturated *vs* saturated FA were measured here, as well as higher levels of ∑n-3 *vs* ∑n-6 FA, which followed initial expectations. The high level of unsaturation within organism tissues was likely driven by low temperatures and high pressures characteristic of cold and deep-water environments [11, 64], as discussed above. In addition, certain PUFA (e.g. n-3 FA) are known key dietary components that are required by aquatic organisms for optimal health, both in shallow [5] and deeper waters [70]. Such essential FA are, for example, involved in cell synthesis, neural development, somatic growth, membrane function and structure, reproduction, ionic regulation, and immune function in aquatic organisms [5, 28, 60]. In particular, docosahexaenoic acid (DHA, 22:6n-3), eicosapentaenoic acid (EPA, 20:5n-3), and arachidonic acid (ARA, 20:4n-6) are all of primary importance for marine species [5], although the extent to which these essential FA occur within organisms may vary [3, 71]. Typically, ARA occurs in lower proportions than EPA and DHA, due to the availability of these FA as dietary sources. The present study was consistent with the literature; although values of individual FA were not provided in the current study, proportions of n-6 FA were up to 9 times lower (e.g. in the Arthropoda) than those of n-3 FA.

Species of the phyla Chordata and Arthropoda represented the most important reservoir of essential nutrients within the faunal assemblage analyzed. Marine organisms, fish in particular, are known to be a major source of PUFA, such as n-3 FA [28, 72, 73]. Marine species containing higher levels of n-3:n-6 FA, PUFA, and DHA+EPA are hence recommended for human consumption, due to their high nutritional value [32, 72]. Furthermore, as DHA, EPA, and to a lesser extent ARA, are likewise largely required by marine organisms and have to be gained through diet [5], feeding habits of marine organisms might be driven by their nutritional needs. In other words, PUFA and essential FA are required at every trophic level and are highly conserved in marine food webs [73]. However, the transfer of these compounds throughout the food web is uneven, and depends on the biochemical and physiological requirements of each taxon [73]. In the present investigation, taking into account that only certain tissues were analyzed for each taxon (see Material and Methods), the Chordata, Arthropoda, and Mollusca had the largest proportions of n-3 FA, while the Chordata, Arthropoda, Echinodermata, and Mollusca had the highest concentrations of DHA+EPA, and the Mollusca and had the highest levels of PUFA. Since neither eggs nor larvae were sampled here, these results suggest that later life stage representatives (juveniles/adults) of these phyla may all constitute important reservoirs of nutrients. However, the overall lipid content of the Echinodermata and Mollusca was relatively low and, therefore, the provision of PUFA and essential FA from these phyla may be limited. In contrast, the Chordata and Arthropoda presented the highest lipid levels in their tissues and, for the same mass, they hence represent a greater reservoir of nutrients than Mollusca. The lack of any significant correlation between total lipid content and wet mass strengthens this result. At the species level, the fish *Coryphaenoides rupestris* and *Gaidropsarus ensis*, as well as the crustacean *Notostomus robustus*, presented the largest levels of essential FA in their tissues, and hence constitute key stores of nutrients among the species analyzed in the Northwest Atlantic. These species are widely distributed in the area [74], although the population of *C*. *rupestris* underwent drastic declines over the last few decades, due to commercial exploitation [74]. As a side note, the vertically migrating species *Lampanyctus* spp., *A*. *pelagica*, *P*. *borealis*, and *N*. *robustus*, included within the Chordata and Arthropoda, were also characterized by high levels of ∑Sat. Since ∑Sat are nutritionally important as a source of energy to consumers [75], these migrating species may play a key role in enhancing the transfer of both essential nutrients and energy between shallow and deeper ecosystems.

Because of the small amount of samples required and the value of the information provided, lipid analysis has supported the investigation of still-poorly-known deep-sea fauna and ecosystems of different oceanic regions, such as the Northeast Pacific [15, 16], Northeast Atlantic [17], and Antarctic [76]. The present study extends this dataset to deep-sea taxa of the Northwest Atlantic and additionally highlights some important findings: i) the wide range of total lipid content and composition suggests a great diversity across deep-sea taxa in terms of energy allocation strategies, which were partly associated with diversified deep-sea adaptations (e.g. migratory behaviors, buoyancy and metabolic needs), and with a variable food supply in the deep sea; ii) the type and amount of energy storage are reflective of habitat (pelagic *vs* demersal), as well as of the type of preferred food sources for certain deep-sea taxa (e.g. WE-rich zooplankton); iii) by modulating ST and FA composition, some species are presumably able to counteract the effect of temperature and pressure along the depth gradient; and finally, iv) representatives of the phyla Chordata and Arthropoda constitute a major reservoir of essential nutrients, and the migrating species included in the two taxa may play a crucial role in transferring these nutrients to deeper food webs.

## Supporting information

S1 Table Proportions of the remaining lipid classes across phyla. Mean proportion % ±se of hydrocarbons (HC), ethyl ethers (EE), methyl esters (ME), ethyl ketones (EK), methyl ketones (MK), glyceryl ethers (GE), alcohols (ALC), diacylglycerols (DAG), and acetone-mobile polar lipids (AMPL) are reported from the phylum containing the highest amounts of lipids to the phylum characterized by the lowest contents.
(DOCX)

S2 Table Lipid class composition across the deep-sea taxa analyzed. Mean proportion % ±sd of phospholipids (PL), free fatty acids (FFA), sterols (ST), triacylglycerols (TAG), wax esters/steryl esters (WE/SE), as well as triacyglycerols to sterols (TAG:ST) and phospholipids to sterols (PL:ST) ratios are reported for each species analyzed in this study.
(DOCX)

S3 Table FA composition across the deep-sea taxa analyzed. Mean value % ±sd of the sum of saturated FA (∑Sat), monounsaturated FA (∑MUFA), polyunsaturated FA (∑PUFA), n-3 FA (∑n-3), n-6 FA (∑n-6), and the sum of docosahexaenoic acid and eicosapentaenoic acids (DHA+EPA) are reported for each species studied. Material and method reports the list of the fatty acids considered in these sums.
(DOCX)

S4 Table Individual fatty acids (%) measured for all the individuals analyzed. (XLSX)
