# Supplementary material for: Functional diversity and nutritional content in a deep-sea faunal assemblage through total lipid, lipid class, and fatty acid analyses
Source: PLoS One. 2018 Nov 12;13(11):e0207395. doi: 10.1371/journal.pone.0207395 (PMC6231680; doi:10.1371/journal.pone.0207395)
Supplement: S1 Table — Mean proportion % ±se of hydrocarbons (HC), ethyl ethers (EE), methyl esters (ME), ethyl ketones (EK), methyl ketones (MK), glyceryl ethers (GE), alcohols (ALC), diacylglycerols (DAG), and acetone-mobile polar lipids (AMPL) are reported from the phylum containing the highest amounts of lipids to the phylum characterized by the lowest contents. (DOCX) [file pone.0207395.s001.docx]

**S1 Table. Proportions of the remaining lipid classes across phyla.** Mean proportion % ±se of hydrocarbons (HC), ethyl ethers (EE), methyl esters (ME), ethyl ketones (EK), methyl ketones (MK), glyceryl ethers (GE), alcohols (ALC), diacylglycerols (DAG), and acetone-mobile polar lipids (AMPL) are reported from the phylum containing the highest amounts of lipids to the phylum characterized by the lowest contents.

| **Phylum** | **HC** | **EE** | **ME** | **EK** | **MK** | **GE** | **AL** | **DAG** | **AMPL** |
| --- | --- | --- | --- | --- | --- | --- | --- | --- | --- |
| Chordata | 1.4±0.1 | 0.5±0.3 | 0.1±0.1 | 1.0±0.3 | 1.9±0.4 | 2.3±1.0 | 4.1±0.6 | 0.3±0.1 | 3.5±0.3 |
| Arthropoda | 2.3±0.3 | 0.3±0.1 | 0.8±0.4 | 1.3±0.5 | 1.8±1.0 | - | 3.1±1.3 | 0.0±0.0 | 2.2±0.6 |
| Echinodermata | 1.9±0.4 | 3.0±0.7 | 0.2±0.1 | 1.5±0.6 | 5.7±1.4 | 1.3±0.9 | 1.6±0.7 | 1.0±0.4 | 2.1±0.5 |
| Annelida | 0.7±0.3 | 0.6±0.3 | 0.7±0.4 | 1.1±0.7 | - | 0.4±0.4 | 2.3±2.3 | - | 2.5±1.5 |
| Cnidaria | 2.9±0.5 | 4.0±1.1 | 1.7±0.9 | 1.4±0.6 | 2.0±1.1 | 0.8±0.3 | 2.6±0.5 | 0.1±0.1 | 5.7±1.5 |
| Mollusca | 0.8±0.2 | - | 0.0±0.0 | 0.3±0.2 | 0.0±0.0 | - | - | - | 0.1±0.1 |
| Porifera | 1.7±0.4 | 4.8±1.5 | 1.5±0.5 | 0.4±0.2 | 0.1±0.1 | - | 0.3±0.1 | 0.3±0.2 | 1.3±0.3 |
| Sipuncula | 2.6±1.6 | - | 3.6±2.8 | - | - | - | - | - | - |
