# Supplementary material for: Functional diversity and nutritional content in a deep-sea faunal assemblage through total lipid, lipid class, and fatty acid analyses
Source: PLoS One. 2018 Nov 12;13(11):e0207395. doi: 10.1371/journal.pone.0207395 (PMC6231680; doi:10.1371/journal.pone.0207395)
Supplement: S2 Table — Mean proportion % ±sd of phospholipids (PL), free fatty acids (FFA), sterols (ST), triacylglycerols (TAG), wax esters/steryl esters (WE/SE), as well as triacyglycerols to sterols (TAG:ST) and phospholipids to sterols (PL:ST) ratios are reported for each species analyzed in this study. (DOCX) [file pone.0207395.s002.docx]

**S2 Table. Lipid class composition across the deep-sea taxa analyzed.** Mean proportion % ±sd of phospholipids (PL), free fatty acids (FFA), sterols (ST), triacylglycerols (TAG), wax esters/steryl esters (WE/SE), as well as triacyglycerols to sterols (TAG:ST) and phospholipids to sterols (PL:ST) ratios are reported for each species analyzed in this study.

| **Phylum** | **Taxon** | **PL** | **FFA** | **ST** | **TAG** | **WE/SE** | **TAG:ST** | **PL:ST** |
| --- | --- | --- | --- | --- | --- | --- | --- | --- |
|  |  | %±sd | %±sd | %±sd | %±sd | %±sd | Mean±sd | Mean±sd |
| **Chordata** |  |  |  |  |  |  |  |  |
|  | Actinopterygii |  |  |  |  |  |  |  |
|  | *A. bairdii* | 23.8±29.9 | 24.1±5.2 | 7.2±7.2 | 29.1±41.1 | - | 26.8 | 2.4±1.7 |
|  | *A. cornuta* | 10.4±5.7 | 13.8±4.0 | 5.3±2.0 | 51.4±8.3 | - | 11.7±7.6 | 1.9±0.4 |
|  | *A. rostrata* | 22.1±18.0 | 50.3±17.2 | 11.1±13.7 | - | - | - | 7.5±7.4 |
|  | *A. risso* | 17.4±7.3 | 10.2±9.2 | 6.2±1.2 | 7.52±0.0 | 23.1±32.7 | 1.2±0.2 | 3.0±1.7 |
|  | *B. euryops* | 19.2±8.7 | 20.6±7.1 | 5.4±2.9 | 40.0±27.8 | - | 10.4±10.8 | 3.7±0.4 |
|  | *B. macrolepis* | 6.5±0.2 | 37.8±17.9 | 10.6±15.1 | 29.9±1.4 | - | 1.4 | 0.3 |
|  | *B. antarcticus* | 23.1±22.3 | 12.5±2.6 | 10.6±10.0 | 20.3±24.1 | 17.2±21.0 | 9.1±1.3 | 2.1±2.0 |
|  | *C. macropus* | 2.0 | 7.2 | 5.6 | 28.3 | 39.0 | 5.1 | 0.4 |
|  | *C. sloani* | 11.1±13.1 | 17.7±12.3 | 7.7±5.8 | 50.8±20.6 | 0.1±0.3 | 12.0±9.8 | 1.8±2.0 |
|  | *C. niger* | 3.9±0.8 | 7.4±2.3 | 1.7±1.3 | 82.9±6.2 | - | 69.5 | 3.0±1.3 |
|  | *C. rupestris* | 38.1±7.1 | 31.4±8.6 | 12.0±3.7 | 9.70±10.5 | 0.1±0.2 | 0.9±1.1 | 3.3±1.1 |
|  | *C. microps* | 36.6±16.6 | 26.2±29.0 | 5.70±8.1 | 1.21±1.7 | - | 0.2 | 4.2 |
|  | *C. thomsonii* | 3.1 | 12.6 | 6.8 | 68.7 | - | 10.1 | 0.5 |
|  | *C. microdon* | 12.5±6.8 | 14.8±4.4 | 9.1±0.3 | 52.6±16.3 | 0.2±0.3 | 5.8±1.6 | 1.4±0.8 |
|  | *G. ensis* | 18.3±6.6 | 54.1±5.2 | 17.5±2.9 | 1.5±3 | 0.2±0.4 | 0.3 | 1.1±0.5 |
|  | *G. cynoglossus* | 28.4±3.6 | 46.3±14.1 | 4.9±8.6 | - | - | - | 2 |
|  | *H. mollis* | 64.6 | 13.9 | 19.5 | - | - | - | 3.3 |
|  | *L. speculigera* | 28.8 | 3.5 | 0.8 | 8.4 | 49.9 | 11.0 | 37.8 |
|  | *Lampanyctus* spp. | 12.6±6 | 12.3±2.4 | 3.8±2.4 | 11.5±3.7 | 40.8±1.7 | 4.0±2.3 | 3.6±0.5 |
|  | *L. eques* | 37.7 | 39.7 | 15.3 | - | - | - | 2.5 |
|  | *M. berglax* | 24.8±6.9 | 45.8±65.7 | 20.7±4.0 | - | - | - | 1.3±0.6 |
|  | *M. atlantica* | 13.1±1.2 | 16±0.9 | 5.7±2.0 | 48.2±3.1 | - | 9.1±3.8 | 2.5±1.1 |
|  | *M. niger* | 2.7±2.3 | 6.7±9.4 | 1.2±1.7 | 41.4±54.9 | - | 33.8 | 0.4 |
|  | *M. johnsonii* | 10.4 | 23.3 | 18 | 28.4 | - | 1.6 | 0.6 |
|  | *Myctophum* sp. | 7.3 | 12.2 | 4.5 | 65.1 | - | 14.5 | 1.6 |
|  | *N. bairdii* | 16.2±7.6 | 32.0±18.2 | 15.1±0.6 | 12.9±15.5 | - | 0.9±1.1 | 1.1±0.5 |
|  | *N. chemnitzii* | 4.4±5.9 | 22.7±1.0 | 10.0±1.8 | 48.7±5.3 | 0.4±0.6 | 5.0±1.4 | 0.5±0.7 |
|  | *Notoscopelus* spp. | 9±4.8 | 9.9±1.1 | 2.4±1.4 | 71.5±5.9 | - | 36.3±23.3 | 3.8±0.2 |
|  | *O. macrosteus* | 44.2 | 3.6 | 24.2 | 1.0 | - | 0.0 | 1.8 |
|  | *P. rissoanus* | 11.0±1.6 | 12.4±3.5 | 5.8±1.8 | 60.3±17.4 | 0.9±0.1 | 11.5±6.6 | 2.0±0.3 |
|  | *R. hippoglossoides* | 24.8 | 9.3 | 1.2 | 56.6 | - | 49.2 | 21.5 |
|  | *S. opisthopterus* | 14.9±0.9 | 28.7±7.3 | 10.1±2.2 | 31.8±4.9 | - | 3.2±0.2 | 1.5±0.4 |
|  | *S. mentella* | 33.1±2.6 | 24.4±0.6 | 13.7±1.7 | 12.0±7.2 | - | 0.9±0.6 | 2.5±0.4 |
|  | *S. beanii* | 39.6±18.5 | 17.8±7.3 | 13.1±3.6 | - | - | - | 3.5±2.6 |
|  | *S. kaupii* | 7.7±3.0 | 10.3±1.9 | 4.3±0.6 | 68.1±5.0 | - | 16.0±3.1 | 1.7±0.4 |
|  | *T. murrayi* | 37.1±6.9 | 42.2±5.1 | 17.2±0.9 | - | - | - | 2.2±0.5 |
|  | *X. copei* | 3.8±3.9 | 14.8±2.6 | 7.7±3 | 64.1±7.3 | - | 9.6±4.6 | 0.6±0.8 |
|  | Ascidiacea |  |  |  |  |  |  |  |
|  | Ascidiacea sp 1 | 60.9±22.8 | 1.8±2.5 | 31.4±13.3 | - | - | - | 2.3±1.7 |
|  | Ascidiacea sp 4 | 64.7±4.8 | 7.4±1.5 | 23.7±9.5 | 2.3±3.2 | - | 0.3 | 3.0±1.4 |
|  | *Didemnum* sp. | 20.6 | 27.8 | 35.6 | - | - | - | 0.6 |
|  | *E. vitreum* | 15.6 | 19.8 | 54.4 | - | - | - | 0.3 |
|  | Chondrichthyes |  |  |  |  |  |  |  |
|  | *A. jenseni* | 80.5 | 1.4 | 15.9 | - | - | - | 5.1 |
|  | *A. profundorum* | 68.3±20.5 | 8±3.9 | 8.5±1.2 | 7.5±13 | 0.0±0.1 | 2.4 | 8.3±3.2 |
|  | *C. fabricii* | 80.3±0.9 | 1.8±0.7 | 7.4±1.1 | 0.1±0.1 | 0.1±0.1 | 0.0 | 11.0±1.8 |
|  | *M. senta* | 50.3 | 3.5 | 14.3 | 3.1 | - | 0.2 | 3.5 |
|  | *R. fyllae* | 38.8±5.6 | 3.5±3.4 | 17±8.6 | 3.4±2.8 | 5.2±5.9 | 0.4±0.3 | 3.0±2.3 |
| **Arthropoda** | |  |  |  |  |  |  |  |
|  | Hexanauplia |  |  |  |  |  |  |  |
|  | *A.* *michelottianum* | 40.5±9.4 | 18.2±5.7 | 13.1±4.7 | 17.5±7.1 | 0.2±0.3 | 1.5±1.1 | 3.4±1.4 |
|  | Malacostraca |  |  |  |  |  |  |  |
|  | *A. pelagica* | 7.8±4.2 | 16.6±12.3 | 13.9±9.1 | 0.3±0.4 | 40.9±15.3 | 0.1 | 0.8±0.8 |
|  | Anonyx sp 1 | 22.5 | 4.3 | 1.8 | 5.0 | 56.8 | 2.8 | 12.7 |
|  | Anonyx sp 2 | 22.1 | 16.5 | 0.9 | 8.7 | 41.8 | 9.4 | 23.8 |
|  | *G. zoea* | 20.2±14.7 | 12.8±3.1 | 8.0±0.1 | - | 38.4±13.3 | - | 2.5±1.8 |
|  | *M. tenuimana* | 25.8 | 26.8 | 14.4 | 30.8 | 0.0 | 2.1 | 1.8 |
|  | *M. curvirostra* | 43.5±22.7 | 13.4±4.8 | 16.9±12.5 | 21.5±37.2 | - | 15.9 | 3.4±1.6 |
|  | *N. robustus* | 31.3 | 33.0 | 26.6 | - | 6.2 | - | 1.2 |
|  | *P. borealis* | 7.2±5.0 | 39.5±13.2 | 23.7±6.6 | - | - | - | 0.3±0.3 |
|  | *P. tarda* | 22.1±18.6 | 41.4±12.7 | 17.1±14.2 | 0.2±0.4 | - | 0.0 | 2.9±2.9 |
|  | *S. hystrix* | 59.5±5.5 | 20.9±3.4 | 13.5±1.6 | 0.5±0.8 | - | 0.1±0.1 | 4.5±0.9 |
|  | *S. sculpta* | 44.5±27.9 | 22.5±16.5 | 24.4±11.1 | - | - | - | 2.3±1.7 |
|  | *T. libellula* | 19.1 | 29.7 | 12.4 | 12.6 | 18.6 | - | - |
|  | Pycnogonida |  |  |  |  |  |  |  |
|  | *Nymphon* spp. | 37.0±27.0 | 33.5±21.4 | 12.3±4.3 | 11.2±3.0 | - | 1.0±0.2 | 4.2±4.8 |
| **Echinodermata** | |  |  |  |  |  |  |  |
|  | Asteroidea |  |  |  |  |  |  |  |
|  | *A. americanus* | 73.6±9.8 | 2.2±1.3 | 9.0±1.9 | 1.1±1.0 | 0.1±0.2 | 0.2±0.0 | 8.3±1.1 |
|  | *Brisingida* spp. | 33.1±37.4 | 12.3±13.0 | 6.8±3.4 | 11.1±12.1 | - | 2.4±3.0 | 4.0±3.5 |
|  | *Cheiraster* sp. | 5.8 | 10.5 | 24 | - | - | - | 0.2 |
|  | *C. crispatus* | 40.5±5.7 | 17.7±5.9 | 20.2±0.9 | - | - | - | 2.0±0.3 |
|  | *F. microspina* | 53.3 | 10.9 | 6.1 | 7.4 | - | 1.2 | 8.7 |
|  | *L. arcticus* | 54.9±7.3 | 12.3±1.1 | 16.6±3.4 | 8.1±12.2 | - |  | 3.3±0.3 |
|  | *M. bairdi* | 39±21.3 | 25.7±15.6 | 22.2±3.6 | 0.5±0.8 | - | 0.1 | 1.9±1.3 |
|  | *M. sol* | 67.0 | 9.1 | 7.6 | 1.1 | - | 0.2 | 8.9 |
|  | *P. andromeda* | 63.6 | 9.0 | 8.2 | 3.2 | - | 0.4 | 7.8 |
|  | *Z. fulgens* | 32.0±14.6 | 13.4±1.1 | 24.5±15.2 | 3.9±6.8 | - | 1.5 | 2.5±2.9 |
|  | Echinoidea |  |  |  |  |  |  |  |
|  | *B. fragilis* | 53.0 | 11.9 | - | 1.6 | - | - | - |
|  | *P. placenta* | 36.8±17.7 | 22.5±12.2 | 15.5±4.0 | 13.5±5.4 |  | 0.9±0.5 | 2.6±1.6 |
|  | *S. pallidus* | 43.0±7.0 | 17.9±2.0 | 18±3.2 | 5.4±4.4 | 0.4±0.6 | 0.3±0.2 | 2.4±0.0 |
|  | Ophiuroidea |  |  |  |  |  |  |  |
|  | *Gorgonocephalus* sp. | 45.8 | 11.7 | 5.5 | 5.2 | - | 0.9 | 8.3 |
|  | *O. aculeata* | 38.6±6 | 13.9±8.7 | 5.8±0.9 | 27.9±5.8 | 0.5±0.1 | 4.8±0.3 | 6.8±2.0 |
|  | *O. glacialis* | 32.1±43.6 | 18.8±15.3 | 5.7±8.0 | 29.5±26.6 | - | 4.3 | 0.1 |
|  | *O. sarsii* | 61.5±9.6 | 14.6±9.5 | 17.7±1.5 | - | - | - | 3.5±0.7 |
| **Annelida** |  |  |  |  |  |  |  |  |
|  | Polychaeta |  |  |  |  |  |  |  |
|  | *A. succinea* | 17.6 | 26.2 | 12.4 | 11.3 | 20.7 | 0.9 | 1.4 |
|  | *L. filicornis* | 43.5 | 15.7 | 18.3 | 10 | 6.1 | 0.5 | 2.4 |
|  | Nereididae sp 2 | 46.9 | 13.1 | 13.2 | 18.2 | 4.5 | 1.4 | 3.5 |
|  | Polychaeta sp 1 | 55.7 | 8.3 | 33.3 | 1.4 | 0.6 | 0.0 | 1.7 |
|  | Polynoidae sp 1 | 30.8 | 42.7 | - | - | - | - | - |
|  | Polynoidae sp 2 | 54.7±7.1 | 18.4±4.7 | 25.5±1.9 | - | - | - | 2.2±0.4 |
|  | Polynoidae sp 3 | 38.5 | 6.6 | 14.2 | 20.2 | - | 1.4 | 2.7 |
|  | *Prionospio* sp. | 1.7 | 45.3 | 51.3 | - | - | 0.0 | 0.0 |
| **Cnidaria** |  |  |  |  |  |  |  |  |
|  | Anthozoa |  |  |  |  |  |  |  |
|  | *A. arbuscula* | 9.5±0.6 | 35.5±20.2 | 13.6±3.7 | 6.9±9.7 | 12.9±1.6 | 0.6±0.9 | 0.7±0.2 |
|  | *A. cristata* | 29.5 | 21.4 | 16.7 | - | - | - | 1.8 |
|  | *A. aurelia* | 18.3 | 34.8 | 26.9 | - | 13.8 | - | 0.7 |
|  | *A. callosa* | 60.1 | 8.6 | 18.4 | - | - | - | 3.3 |
|  | *A. agaricus* | 30.8±18.5 | 14.6±5.1 | 12.3±2.4 | 7.4±12.8 | 15.2±7.7 | 2.1 | 2.4±1.4 |
|  | *Anthomastus* sp. | 46.4 | 12.1 | 14.9 | 4.2 | 8.5 | 0.3 | 3.1 |
|  | *A. grandiflorum* | 17.9 | 42.5 | 7.6 | 5.6 | 7.6 | 0.7 | 2.4 |
|  | *D. florida* | 34.4 | 5.3 | 8.4 | 2.3 | 1.8 | 0.3 | 4.1 |
|  | *F. alabastrum* | 26.7±2.4 | 31.9±6.2 | 11.3±0.6 | 4.0±0.3 | 8.5±1.7 | 0.4±0.0 | 2.4±0.3 |
|  | *Funiculina* sp. | 38.8 | 24.5 | 11.5 | 3.1 | 7.8 | 0.3 | 3.4 |
|  | *P. arborea* | 29.7 | 7.7 | 6.3 | 2.2 | 24.4 | 0.4 | 4.7 |
|  | *P. aculeata* | 27.7±1.5 | 24.3±12.3 | 12.9±2.4 | 6.6±2.7 | 10.4±3.2 | 0.5±0.1 | 2.2±0.4 |
|  | *P. grandis* | 15.1±9.9 | 17.1±0.5 | 8±1.5 | 12.0±3.8 | 9.9±1.7 | 1.6±0.8 | 1.8±0.9 |
|  | *Umbellula* sp. | 15.4 | 13.1 | 6.8 | 4.9 | 32.7 | 0.7 | 2.3 |
|  | Scyphozoa |  |  |  |  |  |  |  |
|  | *A. wyvillei* | 44.7±24.0 | 8.6±0.6 | 12.9±3.0 | - | 15.0±5.7 | - | 3.8±2.7 |
|  | *P. periphylla* | 27.8±29.8 | 14.6±5.3 | 10.3±4.1 | 12.4±17.5 | 25.2±18.0 | 1.7±2.4 | 2.3±2.0 |
| **Mollusca** |  |  |  |  |  |  |  |  |
|  | Cephalopoda |  |  |  |  |  |  |  |
|  | *B. arcticus* | 82.7±1.7 | 4.7±1.1 | 11.9±1.3 | - | - | - | 7.0±0.9 |
|  | *B. bairdii* | 73.0 | 6.7 | 19.5 | - | - | - | 3.7 |
|  | Cephalopoda sp 1 | 67.6 | 16.7 | 13.7 | - | - | - | 4.9 |
|  | Cephalopoda sp 2 | 80.9 | 7.6 | 11.0 | - | - | - | 7.3 |
|  | *C. veranii* | 85.9 | 0.5 | 13.5 | - | - | - | 6.4 |
|  | *I. coindetii* | 69.5±9.8 | 15.4±4.3 | 11.5±2.9 | 2.5±3.0 | - | 0.3±0.2 | 6.5±2.8 |
|  | *N. caroli* | 53.7 | 19.7 | 22.3 | 1.8 | - | 0.1 | 2.4 |
|  | *R. megaptera* | 73.7 | 8.0 | 17.4 | - | - | - | 4.2 |
|  | *S. syrtensis* | 63.4±14.8 | 16.5±6 | 17.5±5.9 | - | - | - | 4.2±2.4 |
|  | Gastropoda |  |  |  |  |  |  |  |
|  | *A. occidentalis* | 48.2 | 27.7 | 22.7 | 0.9 | - | 0.0 | 2.1 |
|  | *Buccinum* sp. | 57.2±13.7 | 22.5±8.6 | 19.9±5.3 | - | - | - | 3.1±1.6 |
|  | *Colus* spp. | 55.7±12.4 | 22.0±11.3 | 21.0±3.9 | - | - | - | 2.7±1.0 |
|  | *N. despecta* | 59.7 | 15.1 | 23.6 | - | - | - | 2.5 |
| **Porifera** |  |  |  |  |  |  |  |  |
|  | Demospongiae |  |  |  |  |  |  |  |
|  | *Cliona* sp. | 70.6 | 12.9 | 12.8 | 0.5 | - | 0.0 | 5.5 |
|  | *C. cranium* | 60.5±4.5 | 15.3±0.6 | 16.0±2.9 | 1.3±1.8 | - | 0.1±0.1 | 3.9±0.8 |
|  | *Geodia* sp. | 74.8 | 10.2 | 10.2 | - | - | - | 7.3 |
|  | *Haliclona* sp. | 56.6±14.4 | 27.3±8.9 | 13.6±4.7 | - | - | - | 4.6±2.7 |
|  | *H.* *carteri* | 24.3 | 7.4 | 32.9 | 8.3 | - | 0.3 | 0.7 |
|  | *Histodermella* sp. | 39.3 | 24 | 20.3 | 4.5 | 7.5 | 0.2 | 1.9 |
|  | *I. piceum* | 65.6 | 7.5 | 18.3 | 3.9 | - | 0.2 | 3.6 |
|  | *M. lingua* | 47.4 | 14.0 | 22.2 | 0.8 | - | 0.0 | 2.1 |
|  | *Phakellia* sp. | 49.9 | 9.7 | 20 | 2.7 | - | 0.1 | 2.5 |
|  | *Polymastia* spp. | 18.2±20.8 | 29.8±6.9 | 21.7±1.71 | 11.5±9.8 | 11.7±3.08 | 0.5±0.4 | 0.9±1.0 |
|  | *P. hemisphaerica* | 36.8 | 18.0 | 17.9 | 7.4 | 13.1 | 0.4 | 2.1 |
|  | *Stelletta* sp. | 48.0 | 31.9 | 13.3 | - | - | - | 3.6 |
|  | *S. ponderosus* | 28.4 | 19.1 | 25.7 | 7.2 | 8.6 | 0.3 | 1.1 |
|  | *T. semisuberites* | 37.4 | 24.9 | 15.8 | 11.2 | 6.8 | 0.7 | 2.4 |
|  | *T. muricata* | 37.7±20.5 | 16.0±3.3 | 21.8±6.84 | 10.8±5.8 | - | 0.5±0.1 | 2.1±1.6 |
|  | Hexactinellida |  |  |  |  |  |  |  |
|  | *Euplectella* sp. | 32.7±3.7 | 11.1±6.1 | 9.9±5.72 | 8.5±8.1 | 10.5±14.8 | 1.3±1.6 | 4.1±2.7 |
|  | Hexactinellida sp 1 | 69.5 | 13.1 | 13.3 | - | - | - | 5.2 |
| **Sipuncula** |  |  |  |  |  |  |  |  |
|  | Sipunculidea |  |  |  |  |  |  |  |
|  | Sipunculidea sp 1 | 69.2 | 7.9 | 21.1 | - | - | - | 3.3 |
|  | Sipunculidea sp 2 | 36.4 | 2.3 | 50.7 | - | - | - | 0.7 |
