# Supplementary material for: Functional diversity and nutritional content in a deep-sea faunal assemblage through total lipid, lipid class, and fatty acid analyses
Source: PLoS One. 2018 Nov 12;13(11):e0207395. doi: 10.1371/journal.pone.0207395 (PMC6231680; doi:10.1371/journal.pone.0207395)
Supplement: S3 Table — Mean value % ±sd of the sum of saturated FA (∑Sat), monounsaturated FA (∑MUFA), polyunsaturated FA (∑PUFA), n-3 FA (∑n-3), n-6 FA (∑n-6), and the sum of docosahexaenoic acid and eicosapentaenoic acids (DHA+EPA) are reported for each species studied. Material and method reports the list of the fatty acids considered in these sums. (DOCX) [file pone.0207395.s003.docx]

**S3 Table. FA composition across the deep-sea taxa analyzed.** Mean value ±sd of the sum of saturated FA (∑Sat), monounsaturated FA (∑MUFA), polyunsaturated FA (∑PUFA), n-3 FA (∑n-3), n-6 FA (∑n-6), and the sum of docosahexaenoic acid and eicosapentaenoic acids (DHA+EPA) are reported for each species studied. Material and method reports the list of the fatty acids considered in these sums.

| **Phylum** | **Taxon** | **n** | **∑Sat** | **∑MUFA** | **∑PUFA** | **∑n-3** | **∑n-6** | **DHA+EPA** |
| --- | --- | --- | --- | --- | --- | --- | --- | --- |
|  |  |  | %±sd | %±sd | %±sd | %±sd | %±sd | g per 100 g wm±sd |
| **Chordata** |  |  |  |  |  |  |  |  |
|  | Actinopterygii |  |  |  |  |  |  |  |
|  | *A. bairdii* | 2 | 29.3±16.7 | 43.6±20.1 | 26.5±3.7 | 22.9±5.4 | 1.8±0.3 | 0.5±0.3 |
|  | *A. cornuta* | 3 | 14.6±3.8 | 74.1±6.2 | 10.7±2.2 | 7.4±3.1 | 1.4±0.3 | 0.7±0.2 |
|  | *A. rostrata* | 3 | 29.1±4.8 | 17.1±1.8 | 53.6±3.1 | 49.4±3.6 | 2.9±1.0 | 0.1±0.0 |
|  | *A. risso* | 2 | 17.8±6.8 | 37.5±10.5 | 40.4±5.4 | 30.4±8.9 | 2.4±0.9 | 0.6±0.4 |
|  | *B. euryops* | 2 | 20.2±1.4 | 51.5±4.3 | 27.6±5.9 | 23.6±6.1 | 2.2±0.6 | 0.3±0.1 |
|  | *B. macrolepis* | 2 | 19.0±3.3 | 40.7±7.2 | 39.9±3.9 | 35.7±4.4 | 3.0±0.1 | 0.1±0.0 |
|  | *B. antarcticus* | 4 | 17.1±4.7 | 48.9±17.5 | 33.2±13.5 | 27.2±11.2 | 2.6±0.9 | 0.2±0.1 |
|  | *C. macropus* | 1 | 1.1 | 7.2 | 51.8 | 19.1 | 2.5 | 0.1 |
|  | *C. sloani* | 6 | 18.2±3 | 60.0±8.5 | 20.9±6.8 | 17.3±7 | 2.2±0.3 | 0.1±0.1 |
|  | *C. niger* | 3 | 18.4±1.7 | 65.6±5.3 | 15.3±3.7 | 11.6±3.7 | 1.6±0.1 | 4.1±2.4 |
|  | *C. rupestris* | 3 | 23.5±2.7 | 28.0±8.8 | 47.1±6.1 | 43.5±5.7 | 1.8±0.6 | 0.1±0.0 |
|  | *C. microps* | 2 | 21.3±2.1 | 28.0±6.2 | 50.3±3.9 | 38.8±2.8 | 10.9±7.2 | 0.2±0.2 |
|  | *C. thomsonii* | 1 | 11.7 | 73.1 | 14.4 | 10.0 | 2.7 | 0.1 |
|  | *C. microdon* | 2 | 18.4±0.8 | 59.5±1.1 | 21.3±1.9 | 17.3±1.4 | 2.1±0.2 | 0.3±0.1 |
|  | *G. ensis* | 4 | 24.1±1.8 | 20.6±2.6 | 54.9±4 | 51.1±2.8 | 3.2±1.1 | 0.1±0.0 |
|  | *G. cynoglossus* | 3 | 32.6±12.0 | 17.8±2.3 | 48.8±10.1 | 36.3±11.7 | 9.2±0.9 | 0.1±0.0 |
|  | *H. mollis* | 1 | 29.9 | 22.4 | 47.5 | 43.8 | 3.2 | 0.1 |
|  | *L. speculigera* | 1 | 15.0 | 44.3 | 38.6 | 28.9 | 2.2 | 0.8 |
|  | *Lampanyctus* spp. | 4 | 13.9±4.4 | 55.9±14.3 | 28.7±9.4 | 24.1±10.1 | 1.5±0.8 | 0.4±0.2 |
|  | *L. eques* | 1 | 33.3 | 13.8 | 52.6 | 49 | 3.3 | 0.2 |
|  | *M. berglax* | 5 | 35.0±8.1 | 13±1.5 | 51.5±7.8 | 44.7±6.5 | 6.3±1.8 | 0.1±0.0 |
|  | *M. atlantica* | 2 | 24.8±1.0 | 47.5±1.3 | 26.7±2.1 | 22.8±1.7 | 2.2±0.2 | 0.8±0.1 |
|  | *M. niger* | 2 | 20.5±1.1 | 58.6±3.5 | 19.9±2.4 | 15.8±2.5 | 2.1±0.1 | 0.7±0.1 |
|  | *M. johnsoni* | 1 | 16.4 | 60.9 | 22.3 | 18.5 | 2.7 | 0.1 |
|  | *Myctophum* sp. | 1 | 23.8 | 46.1 | 29.0 | 24.4 | 2.0 | 3.2 |
|  | *N. bairdii* | 3 | 21.7±3.3 | 20.4±6.0 | 40.4±16.0 | 36.7±15.1 | 2.5±0.9 | 0.1±0.1 |
|  | *N. chemnitzii* | 3 | 21.7±3.3 | 46.6±12.5 | 30±10.9 | 22.6±11.9 | 4.3±2.1 | 0.1±0.0 |
|  | *Notoscopelus* spp. | 2 | 16.2±2.7 | 56.8±14.9 | 26.5±12.2 | 14.1±1.1 | 1.8±1.0 | 2.7±0.4 |
|  | *O. macrosteus* | 1 | 17.1 | 65.1 | 17.3 | 13.2 | 1.3 | 0.0 |
|  | *P. rissoanus* | 3 | 20.1±0.5 | 55.4±3.1 | 23.3±2.9 | 19.2±3.2 | 2.7±0.1 | 0.4±0.2 |
|  | *R. hippoglossoides* | 2 | 19.1±0.7 | 66.2±3.2 | 14.0±3.9 | 10.3±3.3 | 1.5±0.1 | 0.6±0.5 |
|  | *S. opisthopterus* | 2 | 22.0±2.8 | 51.6±0.3 | 25.7±2.3 | 20.0±1 | 2.5±0.3 | 1.6±0.1 |
|  | *S. lepidus* | 1 | 18.8 | 61.1 | 18.8 | 15.5 | 1.5 | 0.5 |
|  | *S. mentella* | 3 | 24.8±6 | 32.4±12.2 | 42.5±6.2 | 38.07±6.2 | 2.7±0.5 | 0.3±0.0 |
|  | *S. beanii* | 3 | 25.8±3.5 | 28.4±3.5 | 45.4±1.9 | 41.6±1.6 | 2.7±0.3 | 0.2±0.1 |
|  | *S. kaupii* | 3 | 13.9±0.8 | 72.6±2.5 | 12.7±1.9 | 9.4±1.9 | 1.6±0.1 | 0.9±0.6 |
|  | *T. murrayi* | 3 | 30.2±7 | 18.9±2.9 | 50.5±4.4 | 44.1±3.6 | 5.5±0.5 | 0.1±0.0 |
|  | *X. copei* | 4 | 22.4±1.1 | 49.0±2.0 | 27.4±1.2 | 21.8±1.3 | 2.5±0.4 | 0.4±0.1 |
|  | Ascidiacea |  |  |  |  |  |  |  |
|  | Ascidiacea sp 1 | 4 | 24.2±7.7 | 46.0±2.9 | 24.4±7.1 | 11.1±4.1 | 8.2±4.4 | 0.0 |
|  | Ascidiacea sp 2 | 1 | 24.5 | 53.8 | 19.4 | 9.7 | 7.6 | 0.0±0.0 |
|  | Ascidiacea sp 3 | 1 | 21.8 | 26.0 | 40.9 | 27.7 | 6.6 | 0.0 |
|  | Ascidiacea sp 4 | 2 | 17.0±0.4 | 35.5±3.6 | 41.4±2.8 | 27.5±1.1 | 12.8±2.1 | 0.0 |
|  | *Didemnum* sp. | 1 | 18.9 | 36.7 | 39.5 | 30.2 | 8.8 | 0.0±0.0 |
|  | *E. vitreum* | 1 | 21.2 | 47.2 | 23.7 | 16 | 4.8 | 0.0 |
|  | Chondrichthyes |  |  |  |  |  |  |  |
|  | *A. jenseni* | 1 | 26.9 | 23.9 | 48.6 | 40.5 | 6.7 | 0.2 |
|  | *A. profundorum* | 3 | 23.9±4.5 | 27.1±6.8 | 48.2±9 | 41.8±8.3 | 4.6±0.9 | 0.2±0.0 |
|  | *C. fabricii* | 2 | 23.3±0.3 | 30.7±0.1 | 45.5±0.5 | 38.2±1.3 | 5.1±1.3 | 0.1±0.0 |
|  | *M. senta* | 1 | 8.0 | 56.0 | 31.9 | 27.0 | 1.5 | 0.1 |
|  | *R. fyllae* | 4 | 25.7±1.8 | 40.6±7.1 | 32.7±5.4 | 25.9±5.1 | 5.1±0.5 | 1.9±1.2 |
| **Arthropoda** | |  |  |  |  |  |  |  |
|  | Hexanauplia |  |  |  |  |  |  |  |
|  | *A.* *michelottianum* | 3 | 16.2±2.2 | 47.1±8.1 | 35.8±7.8 | 32.1±7.7 | 2.1±0.2 | 0.2±0.1 |
|  | Malacostraca |  |  |  |  |  |  |  |
|  | *A. pelagica* | 3 | 8.9±2.4 | 52.6±5 | 35.6±5.8 | 32.5±4.8 | 1.6±0.2 | 0.2±0.1 |
|  | Anonyx sp 1 | 1 | 2.0 | 57.6 | 38.4 | 11.1 | 0.8 | 0.2 |
|  | Anonyx sp 2 | 1 | 5.3 | 55.5 | 26.1 | 7.0 | 0.7 | 0.8 |
|  | *G. zoea* | 3 | 9.3±1.2 | 55.5±10.6 | 31.1±13.3 | 27.0±12.6 | 1.5±0.2 | 0.2±0.1 |
|  | *M. tenuimana* | 1 | 20.0 | 38.1 | 41.5 | 36.4 | 2.2 | 0.0 |
|  | *M. curvirostra* | 3 | 17.5±0.4 | 54.3±11.5 | 27.8±11.2 | 25.1±11.8 | 2.0±0.7 | 0.4±0.4 |
|  | *N. robustus* | 1 | 19.7 | 31.5 | 48.5 | 44.6 | 2.1 | 0.1 |
|  | *P. borealis* | 3 | 23.0±6.4 | 34.3±3.5 | 42.1±8.8 | 37.8±9.3 | 3.4±1 | 0.2±0.0 |
|  | *P. tarda* | 3 | 12.6±8.5 | 41.9±4 | 41.1±2.7 | 36.7±2.1 | 2.7±0.2 | 0.2±0.1 |
|  | *S. hystrix* | 3 | 18.1±1.1 | 38.7±2.7 | 41.5±1.8 | 34.8±1.4 | 4.3±0.5 | 0.2±0.1 |
|  | *S. sculpta* | 3 | 23.8±3.9 | 37.4±5 | 38.7±8.6 | 27.7±14 | 10.1±11.3 | 0.1±0.0 |
|  | *T. libellula* | 1 | 12.5 | 38.0 | 39.2 | 17.9 | 3.1 | 0.1 |
|  | Pycnogonida |  |  |  |  |  |  |  |
|  | *Nymphon* spp. | 6 | 22.0±7.2 | 37.9±5.9 | 38.0±5.0 | 31.2±5.3 | 4.8±1.1 | 0.1±0.1 |
| **Echinodermata** | |  |  |  |  |  |  |  |
|  | Asteroidea |  |  |  |  |  |  |  |
|  | *A. americanus* | 3 | 13.0±1.9 | 42.8±10.3 | 49.8±1.8 | 23.9±1.8 | 22.7±1.6 | 0.3±0.2 |
|  | *Brisingida* spp. | 2 | 7.9±3.6 | 42.2±10.3 | 47.2±10.8 | 27.1±5.1 | 0.9±0.9 | 0.4±0.3 |
|  | *Cheiraster* sp. | 1 | 17.5 | 37.3 | 28.1 | 21.6 | 9.9 | 0.0 |
|  | *C. crispatus* | 3 | 23.3±20.6 | 39.5±11.7 | 38.7±16.5 | 7.1±0.7 | 24.4±18.9 | 0.0±0.0 |
|  | *F. microspina* | 1 | 10.0 | 39.3 | 44.4 | 7.4 | 17.3 | 1.0 |
|  | *L. arcticus* | 3 | 13.2±1.4 | 45.2±1.6 | 42.2±3.6 | 23.5±7.2 | 9.3±2.5 | 0.1±0.0 |
|  | *M. bairdi* | 3 | 6.6±1.6 | 43.2±3.3 | 44.6±4.8 | 13.9±10.5 | 28.8±5.8 | 0.0±0.0 |
|  | *M. sol* | 1 | 12.2 | 48.6 | 60.5 | 8.2 | 29.5 | 0.0 |
|  | *P. andromeda* | 2 | 12.1±1.0 | 31.9±8.3 | 39.5±14.7 | 16.4±5.2 | 28.2±1.1 | 0.4±0.6 |
|  | *Z. fulgens* | 3 | 10.1±4.5 | 52±13.1 | 42.8±12.0 | 23.4±8.0 | 9.3±4.2 | 0.2±0.3 |
|  | Echinoidea |  |  |  |  |  |  |  |
|  | *B. fragilis* | 1 | 27.9 | 40.9 | 45.3 | 33.2 | 11.3 | 0.0 |
|  | *P. placenta* | 3 | 16.5±1.6 | 38.6±10.7 | 36.6±2.7 | 15.6±3.9 | 16.4±1.4 | 0.1±0.0 |
|  | *S. pallidus* | 2 | 20.1±0.3 | 36.7±9.4 | 45.1±4.3 | 21.8±10.8 | 9.9±3.0 | 0.0±0.0 |
|  | Ophiuroidea |  |  |  |  |  |  |  |
|  | *Gorgonocephalus* sp. | 1 | 11.3 | 34.1 | 38.1 | 23.5 | 1.6 | 0.5 |
|  | *O. aculeata* | 2 | 19±0.7 | 46.9±3.0 | 34.1±2.2 | 28.7±5.2 | 3.5±1.4 | 0.2±0.2 |
|  | *O. glacialis* | 2 | 17.7±6.6 | 47.3±0.2 | 26.2±0.2 | 20.4±2.7 | 2.6±1.0 | 0.2±0.0 |
|  | *O. sarsii* | 3 | 18.8±8.3 | 54.8±10.9 | 28.4±0.7 | 14.8±1.9 | 9±0.6 | 0.0±0.0 |
| **Annelida** |  |  |  |  |  |  |  |  |
|  | Polychaeta |  |  |  |  |  |  |  |
|  | *A. succinea* | 1 | 15.5 | 37.5 | 44.5 | 33.2 | 3.9 | 0.2 |
|  | *L. filicornis* | 1 | 23.9 | 34.9 | 40.2 | 19.8 | 2.6 | 0.1 |
|  | Nereididae sp 1 | 1 | 19.5 | 39.5 | 40.2 | 22.4 | 8.4 | 0.1 |
|  | Nereididae sp 2 | 1 | 18.0 | 40.5 | 37.5 | 27.2 | 4.1 | 0.3 |
|  | Polynoidae sp 1 | 1 | 17.8 | 41.9 | 39.6 | 28.9 | 6.0 | 0.1 |
|  | Polynoidae sp 2 | 2 | 21.1±0.7 | 30.3±0.7 | 48.1±0.2 | 38±1.6 | 4.9±0.3 | 0.1±0.0 |
|  | Polynoidae sp 3 | 1 | 29.0 | 46.5 | 22.9 | 14.7 | 2.5 | 0.1 |
|  | *Prionospio* sp. | 1 | 18.0 | 47.6 | 34.2 | 26.2 | 4.4 | 0.1 |
| **Cnidaria** |  |  |  |  |  |  |  |  |
|  | Anthozoa |  |  |  |  |  |  |  |
|  | *A. arbuscula* | 3 | 16.2±1.4 | 44.0±2.4 | 35.3±4 | 20.0±4.4 | 12.3±2.6 | 0.0±0.0 |
|  | *A. cristata* | 2 | 21.2±9.2 | 54.0±9.6 | 17.6±20.6 | 7.2±10.2 | 0.3±0.1 | 0 |
|  | *A. aurelia* | 3 | 21.8±5.1 | 40.1±9.9 | 40.1±3.3 | 18.9±8.3 | 10.3±13.8 | 0.0±0.0 |
|  | *A. callosa* | 3 | 16.4±3.2 | 41.7±17.4 | 39.2±14 | 26.1±9.4 | 6.5±4.3 | 0.0±0.0 |
|  | *A. agaricus* | 3 | 15.6±3.8 | 51.3±11.3 | 27.0±3.0 | 15.8±15.3 | 17.4±0.3 | 0.0±0.0 |
|  | *Anthomastus* sp. | 1 | 15.8 | 48.6 | 49.8 | 12.3 | 8.8 | 0.1 |
|  | *A. grandiflorum* | 1 | 16.1 | 31.9 | 31.0 | 38.4 | 4.2 | 0.6 |
|  | *D. florida* | 1 | 18.0 | 51.6 | 49.1 | 25.7 | 40.4 | 0.0 |
|  | *F. alabastrum* | 2 | 17.9±2.3 | 31.5±9.9 | 41±0.2 | 13.6±8.4 | 19±1.2 | 0.1±0.0 |
|  | *Funiculina* sp. | 1 | 15.2 | 38.3 | 43.4 | 22.0 | 3.3 | 0.2 |
|  | *P. arborea* | 1 | 11.3 | 40.1 | 45.9 | 36.9 | 9.5 | 0.1 |
|  | *P. aculeata* | 3 | 11.9±1.3 | 49.0±8.1 | 35.7±6.6 | 19.9±10.7 | 20.6±4.8 | 0.1±0.1 |
|  | *P. grandis* | 2 | 13±0.6 | 47.6±1.1 | 36.4±1.3 | 22.5±8.6 | 6.3±2.8 | 0.2±0.1 |
|  | *Umbellula* sp. | 1 | 5.1 | 49.0 | 46.6 | 26.8 | 3.2 | 0.4 |
|  | Scyphozoa |  |  |  |  |  |  |  |
|  | *A. wyvillei* | 3 | 24.4±2.3 | 42.1±5.5 | 36.7±4.8 | 27.7±4.1 | 4.9±0.9 | 0.0±0.0 |
|  | *P. periphylla* | 4 | 23.6±2.7 | 46.3±6.3 | 28.7±7.0 | 19.6±4.3 | 2.7±0.7 | 0.0±0.0 |
|  | Scyphozoa sp. | 1 | 18.1 | 49.0 | 25.1 | 21.2 | 2.2 | 0.0 |
| **Mollusca** |  |  |  |  |  |  |  |  |
|  | Cephalopoda |  |  |  |  |  |  |  |
|  | *B. arcticus* | 3 | 26.6±0.7 | 24.4±20.9 | 58.9±3.6 | 37.8±30.1 | 2.5±0.4 | 0.2±0.1 |
|  | *B. bairdii* | 1 | 30.3 | 18.6 | 31.9 | 48.9 | 3.6 | 0.1 |
|  | Cephalopoda sp 1 | 1 | 25.8 | 16.0 | 55.8 | 24.5 | 0.8 | 0.3 |
|  | Cephalopoda sp 2 | 1 | 25.1 | 18.3 | 55.4 | 53.5 | 1.6 | 0.3 |
|  | *C. veranii* | 1 | 25.4 | 37.5 | 54.9 | 24.6 | 2.3 | 0.4 |
|  | *I. coindetii* | 3 | 21.7±0.8 | 18.4±0.9 | 60.3±0.8 | 55.3±3.7 | 1.8±0.7 | 0.4±0.1 |
|  | *N. caroli* | 1 | 24.4 | 17.9 | 60.0 | 55.0 | 1.3 | 0.2 |
|  | *R. megaptera* | 1 | 55.3 | 15.4 | 28.3 | 55.5 | 0.5 | 0.1 |
|  | *S. syrtensis* | 3 | 34.7±18.3 | 19.3±3.5 | 45.9±16.3 | 50.5±3.8 | 2.8±0.3 | 0.1±0.0 |
|  | Gastropoda |  |  |  |  |  |  |  |
|  | *A. occidentalis* | 1 | 15.5 | 16.7 | 59.8 | 22.7 | 10.0 | 0.1 |
|  | *Buccinum* sp. | 3 | 21.8±1.0 | 21.8±1.8 | 55.9±1.0 | 38.9±1.6 | 5.7±0.8 | 0.1±0.0 |
|  | *Colus* spp | 3 | 21.6±0.5 | 20.7±1.0 | 56.9±1.7 | 36.1±4.6 | 16.7±5.4 | 0.0±0.0 |
|  | *N. despecta* | 1 | 36.9 | 20.4 | 43.8 | 25.0 | 16.6 | 0.0 |
| **Porifera** |  |  |  |  |  |  |  |  |
|  | Demospongiae |  |  |  |  |  |  |  |
|  | *Cliona* sp. | 1 | 10.5 | 18.5 | 9.4 | 13.2 | 0.3 | 0.0 |
|  | *C. cranium* | 3 | 13.3±1.6 | 59.0±4.0 | 5±1.8 | 4.0±4.0 | 0.6±0.3 | 0.0±0.0 |
|  | *Geodia* sp. | 1 | 27.6 | 68.5 | 24.1 | 2.3 | 0.1 | 0.0 |
|  | *Haliclona* sp. | 2 | 23.7±4.7 | 28.6±24.8 | 36±39.8 | 4.4±3.4 | 0.2±0.2 | 0.0 |
|  | *H.* *carteri* | 1 | 13.9 | 53.4 | 10.9 | 4.1 | 0.0 | 0.0 |
|  | *Histodermella* sp. | 1 | 38.3 | 63.6 | 12.0 | 12.0 | 5.2 | 0.1 |
|  | *I. piceum* | 1 | 11.1 | 49.7 | 1.2 | 12.3 | 6.0 | 0.1 |
|  | *Phakellia* sp. | 1 | 29.8 | 73.2 | 4.4 | 29.6 | 2.3 | 0.0 |
|  | *Polymastia* spp. | 2 | 16.8±0.5 | 59.2±8.4 | 29.2±12.2 | 4.9±2.5 | 2.6±3.6 | 0.1±0.1 |
|  | *R. hemisphaerica* | 1 | 55.2 | 40.5 | 11.8 | 26.8 | 2.7 | 0.0 |
|  | *Stelletta* sp. | 1 | 26.4 | 31.2 | 3.3 | 13.8 | 0.0 | - |
|  | *S. ponderosus* | 1 | 13.6 | 70.3 | 33.7 | 0.5 | 4.8 | 0.1 |
|  | *T. semisuberites* | 1 | 21.6 | 42.4 | 18.0 | 30.7 | 6.8 | 0.2 |
|  | *T. muricata* | 4 | 20.2±9.6 | 52.4±14.6 | 25.0±10.1 | 16.1±5.1 | 3.0±2.6 | 0.0±0.0 |
|  | Hexactinellida |  |  |  |  |  |  |  |
|  | *Euplectella* sp. | 1 | 22.6 | 39.1 | 28.9 | 27.6 | 1.8 | 0.1 |
|  | Hexactinellida sp 1 | 1 | 9.0 | 43.2 | 68.1 | 20.9 | 0.2 | 0.0 |
|  | Hexactinellida sp 2 | 1 | 18.4 | 20.5 | 28.7 | 1.0 | 0.2 | 0.0 |
| **Sipuncula** |  |  |  |  |  |  |  |  |
|  | Sipunculidea |  |  |  |  |  |  |  |
|  | Sipunculidea sp 1 | 1 | 23.4 | 65.2 | 50.4 | 4.1 | 12.3 | 0.1 |
|  | Sipunculidea sp 2 | 1 | 29.9 | 24.1 | 17.7 | 19.3 | 4.2 | 0.0 |
